# The global genomic landscape of hypervirulent *Klebsiella pneumoniae* from 1932 to 2021

**DOI:** 10.1002/mlf2.70029

**Published:** 2025-08-24

**Authors:** Xiaoyuan Jiang, Shuangshuang Li, Cuidan Li, Zhe Yin, Fangzhou Chen, Lingfei Hu, Tianyu Lu, Xiaoqiang Liu, Yinyu Wang, Guannan Ma, Xiaoyu Wang, Fei Chen, Dongsheng Zhou

**Affiliations:** ^1^ National Genomics Data Center China National Center for Bioinformation Beijing China; ^2^ Beijing Institute of Genomics Chinese Academy of Sciences Beijing China; ^3^ University of Chinese Academy of Sciences Beijing China; ^4^ State Key Laboratory of Pathogen and Biosecurity Academy of Military Medical Sciences Beijing China; ^5^ Department of Environment and Information Studies Keio University Fujisawa Japan; ^6^ State Key Laboratory of Pathogenesis, Prevention and Treatment of High Incidence Diseases in Central Asia Urumqi China; ^7^ Key Laboratory of Viral Pathogenesis & Infection Prevention and Control (Jinan University), Ministry of Education Guangzhou China

**Keywords:** antimicrobial resistance (AMR), hypervirulent *K. pneumoniae* (hvKp), virulence determinant combinations (VDCs), virulence gene profiles (VGPs), virulence‐related accessory genetic elements (VAGEs)

## Abstract

The global spread of hypervirulent *Klebsiella pneumoniae* (hvKp) poses a serious public health threat. In this study, we conducted genomic epidemiology analysis on 2097 global hvKp isolates, including our 900 isolates sequenced through the Illumina platform (177 of them fully sequenced through PacBio platform), representing the most comprehensive genomic analysis of hvKp to date. Our results identified six dominant clonal groups (CGs), particularly including CG23 and CG258, and 17 major virulence determinant combinations (VDCs) comprising 10 virulence gene profiles (VGPs), four types of virulence plasmids, four ICE*Kp* variants, Tn*7399*, and *all*_island. Each CG harbored distinct advantageous VDCs, indicating strong genomic correlation and co‐evolution. Additionally, the phylogeny and evolutionary history of CG23 and CG258 were characterized in depth. Notably, 41.58% of the 2097 isolates were multidrug‐resistant and 33.29% were carbapenem‐resistant, indicating serious antimicrobial resistance. Overall, our study provides a global genomic landscape of hvKp, emphasizing the genetic basis for their global dissemination and the need for precise prevention and control.

## Impact statement

This study provides the most comprehensive genomic analysis of hvKp, examining 2097 global samples collected over 90 years. We identified six major clonal groups (CGs), and performed an in‐depth analysis of their virulence determinants, including virulence genes, plasmids, ICE*Kp*, Tn*7399*, and *all*_island. The results uncovered strong genomic associations and co‐evolution between CGs and these determinants. Notably, one‐third of the strains were resistant to carbapenem antibiotics, posing serious treatment challenges. These findings offer critical insights into how hvKp spreads and evolves, helping researchers and healthcare professionals better monitor, prevent, and control these dangerous infections worldwide.

## INTRODUCTION

*Klebsiella pneumoniae* belongs to the family *Enterobacteriaceae* and can be classified into classic *K pneumoniae* (cKp) and hypervirulent *K. pneumoniae* (hvKp)^1^, ^2^, ^3^. cKp is a ubiquitous opportunistic pathogen that mainly causes nosocomial infection in immunocompromised population^1^. Compared to cKp, hvKp represents a much more severe threat to public health because hvKp affects individuals with intact immune functions and thus predominantly leads to community‐acquired invasive infections such as liver abscesses and meningitis^2^.

The hypervirulence of hvKp is attributed to the acquisition of multiple exogenous virulence gene loci, primarily including *peg‐344*, *iuc*, *iro*, *rmpA/rmpA2*, *ybt*, *clb*, *mce*, and *all* ^2^, ^4^. *peg‐344* encodes a metabolite transporter specific to hvKp and is essential for the hypervirulence in a pneumonia animal model^5^. Three distinct siderophores, namely, aerobactin (*iuc*), salmochelin (*iro*), and yersiniabactin (*ybt*), are more commonly present in hvKp than cKp, and they, in combination, contribute to the hypervirulence due to their roles in enhancing iron uptake^6^. The two paralogous transcription factors *rmpA* and *rmpA2* upregulate the expression of capsule synthesis (*cps*), a key contributor of the hypermucoviscous phenotype^7^. Colibactin (*clb*) supports the colonization and pathogenesis of hvKp^8^. Microcin E492 (*mce*), an 8‐kDa bacteriocin active against *Enterobacteriaceae*, endows a competitive advantage to hvKp for colonization in colonic environment^2^. The virulence gene locus *all* accounts for allantoin metabolism required for hypervirulence^9^. The above virulence gene loci are commonly located within four different virulence‐related accessory genetic elements (VAGEs), namely, virulence plasmids^10^, ^11^, ICE*Kp* ^12^, ^13^, GIE492^14^, and *all*_island^9^.

Typically, each hvKp isolate contains a virulence plasmid^10^, ^11^, and its curing significantly reduces the hypervirulence^15^, ^16^, ^17^. Virulence plasmids can be assigned into several incompatibility (Inc) groups such as IncFIB, IncHI, IncFII, and IncR^18^. Virulence genes on these plasmids commonly include *peg‐344*, *iuc*, *iro*, and *rmpA/rmpA2* ^15^, ^16^. As shown by a cohort study, the separate presence of these plasmid‐carrying virulence genes can differentiate hvKp from non‐hvKp isolates with over 95% accuracy, while the combined assessment of *peg‐344* and *iucA* increases this accuracy to 98%^19^.

Several important VAGEs are found on the chromosome of hvKp. ICE*Kp*, an integrative and conjugative element (ICE), carries the accessory locus *ybt* or *ybt* + *clb* and includes core modules such as *int* (integrase), *xis* (excisionase), and the type IV secretion system (T4SS) for conjugation^12^, ^13^. At least 22 ICE*Kp* variants have been identified, with ICE*Kp10* being the most prevalent^20^. Additionally, GIE492 (renamed Tn*7399* herein) and *all*_island are two chromosomal virulence islands with conserved gene organization and sequence homology across different hvKp strains^21^. Tn*7399*, an integrative and mobilizable element (IME), encodes integrase but lacks T4SS genes^14^, ^22^. *all*_island, a pathogenicity island, carries the accessory *all* locus but does not encode any known mobilization determinants, making it unassignable to any existing transposons^9^.

Unfortunately, hvKp has shown an alarming evolution toward multidrug resistance (MDR)^23^. In particular, the convergence of hypervirulence and carbapenem resistance (CR) mainly follows two evolutionary paths: hvKp acquires resistance plasmids or modules to evolve into CR‐hvKp, while carbapenem‐resistant *K. pneumoniae* (CRKp) integrates virulence plasmids or modules to evolve into hv‐CRKp^23^, ^24^.

Traditional genotyping studies reveal three dominant clonal groups (CGs) in hvKp: CG23, CG65, and CG86, with two primary capsule locus (KL) types: KL1 and KL2. CG23, the most prevalent hvKp CG, is strongly associated with KL1, while CG65 and CG86 are linked to KL2^2^, ^25^, ^26^. CG258, the most common hv‐CRKp CG, is primarily associated with KL47 and KL64^27^, ^28^, ^29^. However, current genomic studies often focus on specific CGs, such as CG23 or CG258/ST11 (sequence type 11)^21^, ^30^, leaving gaps in understanding the global spread of hvKp and its genetic relationships with genotypes, virulence genes, VAGEs, and antimicrobial resistance (AMR) genes. Thus, more comprehensive genomic studies on global hvKp strains are needed.

In this study, we conducted high‐throughput sequencing of 900 Chinese hvKp isolates, including 177 with complete genome sequencing, and integrated 1197 publicly available hvKp genomes from GenBank, resulting in a total dataset of 2097 hvKp isolates collected between 1932 and 2021. This dataset represents the most comprehensive genomic epidemiological analysis of hvKp to date, providing a global overview of STs/CGs, KLs, virulence gene profiles (VGPs), VAGEs, virulence determinant combinations (VDCs), AMR genes, and the prevalence of MDR‐hvKp and CR‐hvKp/hv‐CRKp. Our findings offer critical insights for the targeted prevention and control of hvKp infections.

## RESULTS

### Geographical and temporal distribution of the 2097 hvKp isolates

**Figure 1:**
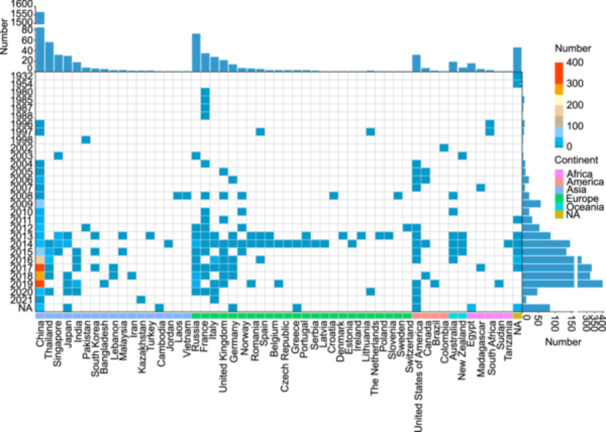
Spatial‐temporal distribution of 2097 hypervirulent *K. pneumoniae* (hvKp). The heatmap shows distribution of the 2097 isolates by year, country, and region. Bar charts above and to the right of the heatmap indicate the total number of isolates per country and per year, respectively. Countries from the same continent are marked with the same color below the heatmap. NA, indicates the isolation year or country for the corresponding strains are not available.

We collected 900 hvKp isolates from 46 hospitals across 15 provinces in China between 2004 and 2019 (Figure S1 and Table S1). These were sequenced using the Illumina platform, with 177 further sequenced for complete genomes on the PacBio platform (Table S1). Together with 1197 hvKp genome sequences from GenBank (as of Dec 31, 2021) (Figure S1 and Table S1), our analysis encompassed a total of 2097 hvKp isolates spanning 52 countries from five continents, with Asia contributing the largest proportion (82.93%, 1739/2097), followed by Europe (10.35%, 217/2097) and the Americas (2.00%, 42/2097) (Figure 1 and Table S1). The isolation period ranged from 1932 to 2021, with a significant increase observed after 2013 (83.69%, 1755/2097) (Figure 1 and Table S1).

### Classification and prevalence of CGs

**Figure 2:**
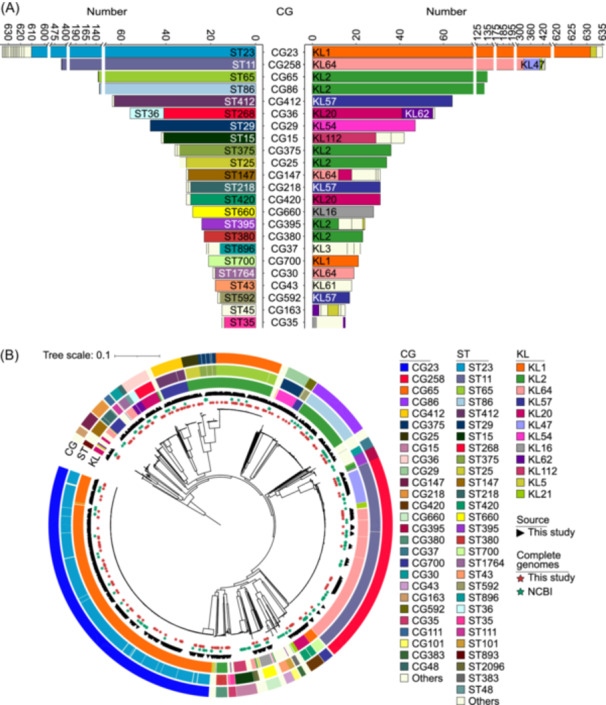
STs, CGs, and KL types, and the ML clustering tree of the 2097 hvKp isolates. (A) Distribution of STs and KL types within major CGs (each *n* ≥ 15). (B) ML phylogenetic tree based on 139,122 core SNPs from the 2097 isolates. *Klebsiella variicola* DSM 15968 was used as the outgroup (not shown). Circles from inner to outer layers represent KL types, STs, and CGs, respectively. Black triangles indicate the 900 hvKp isolates sequenced in this study; red and green stars denote complete genomes sequenced in this study and from GenBank, respectively. CG, clonal group; KL, capsule locus; ML, maximum‐likelihood; SNP, single‐nucleotide polymorphism; ST, sequence type.

As shown by multi locus sequence typing (MLST) analysis, the 2097 hvKp isolates belonged to 139 STs, including 18 novel ones (ST5210 to ST5213 and ST5930 to ST5943), and were further categorized into 82 CGs (Figure 2A and Table S1). The top six CGs accounted for 69.05% of all isolates (CG23: 30.28%; CG258: 20.51%; CG65: 6.44%; CG86: 6.10%; CG412: 3.05%; CG36: 2.67%). The dominant STs within the top six CGs were ST23 (96.22%), ST11 (99.53%), ST65 (99.26%), ST86 (99.22%), ST412 (98.44%), and ST268 (73.21%). KL typing identified 50 different KL types, with KL1 (31.43%) and KL2 (18.26%) being the most dominant, followed by KL64 (17.60%), KL57 (5.39%), KL20 (4.24%), KL47 (3.67%), and KL54 (2.38%) (Figure 2A and Table S1). Each CG was associated with one or two specific KL types (Figure 2A). For instance, the top six CGs were predominantly associated with KL1, KL64 + KL47, KL2, KL2, KL57, and KL20 + KL62.

We then observed a degree of specific geographical and temporal distribution of CGs. Most CGs were predominantly found in Asia, particularly CG23 (29.73%, 517/1739), CG258 (23.92%, 416/1739), and CG65 (7.02%, 122/1739); in contrast, CG23 (35.14%, 91/259), CG86 (9.27%, 24/259), and CG395 (8.88%, 23/259) were more prevalent in Europe and America (Figure S2A). A temporal analysis indicated a significantly increasing trend of CG23 and CG258 (especially CG258) after 2010 (Figure S2B).

### Complex semi‐clonal population structure

We constructed a maximum‐likelihood (ML) clustering tree from the core‐genome single‐nucleotide polymorphisms (SNPs) of the 2097 hvKp isolates (Figure 2B). Isolates within the same CG were clustered together to form a lineage distinct from isolates of other CGs in the ML tree; clonal spread could be found in most CGs, especially the top six CGs, but dramatic genomic diversification was observed among CGs, displaying a complex semi‐clonal population structure of the 2097 hvKp isolates (Figure 2B). Notably, CG23 isolates were on the farthest branch from the root in the ML tree, indicating the most recently differentiated clone; CG258 isolates were on the relatively earlier divergent branches, while all other top four CGs were on the initial divergent branches (Figure 2B). In addition, isolates from numerous smaller CGs (each *n* < 50) were dispersed throughout the ML tree, particularly on the initial divergent branches and between the branches for CG23 and CG258 (Figure 2B). A parallel analysis of our sequenced 900 isolates revealed a similar complex semi‐clonal population structure (Figure S3). Additionally, phylogeny‐independent assessment by using fineSTRUCTURE confirmed that isolates from each CG were consistently clustered (Figure S4), aligning with the lineages in the ML tree.

### Classification and prevalence of VGPs

**Figure 3:**
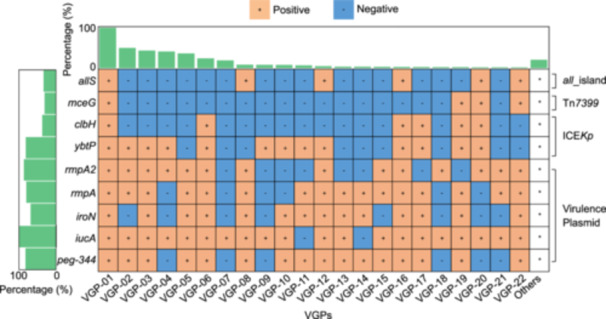
Prevalence of nine key virulence genes and VGPs among 2097 hvKp isolates. The nine key virulence genes are listed on the vertical axis; VGPs are shown on the horizontal axis. Bar charts to the left and top display the positive rates of each virulence gene and VGP, respectively. Five key virulence genes (*peg‐344*, *iucA*, *iroN*, *rmpA*, and *rmpA2*) are typically located on virulence plasmids, while *yptP* + *clbH*, *mceG*, and *allS* are commonly associated with ICEK*p*, Tn*7399*, and *all_*island, respectively. VGPs, virulence gene profiles.

In our analysis of the 2097 hvKp isolates, we focused on the previously reported nine key virulence genes *peg‐344*, *iucA*, *iroN*, *rmpA*, *rmpA2*, *yptP*, *clbH*, *mceG*, and *allS* ^5^, ^9^, ^12^, ^13^, ^14^, ^15^, ^16^, ^19^. The first six genes were found in >50% of the isolates and *iucA* was the most prevalent (96.47%), followed by *rmpA2* (83.79%) and *peg‐344* (79.97%), whereas the last three genes were present in <50% of the isolates (Figure 3 and Table S1). Furthermore, we identified a total of 22 main VGPs (each *n* ≥ 10) in the hvKp isolates, and the most prevalent VGP‐01 (25.27%) contained all nine key virulence genes, followed by VGP‐02 to VGP‐05 accounting for 12.59%, 10.92%, 10.40%, and 9.20%, respectively (Figure 3). In addition, the top six CGs primarily involved the 10 major VGPs (VGP‐01 to VGP‐06, VGP‐08, VGP‐12, VGP‐14, and VGP‐22), comprising 90.33% of the isolates of these six CGs (Figure S5A).

### Classification and prevalence of VAGEs

The four VAGEs, namely, virulence plasmids, ICE*Kp*, Tn*7399,* and *all*_island, were exclusively responsible for the dissemination of the above nine key virulence genes (Figure 3), being consistent with previous research^9^, ^12^, ^13^, ^14^, ^15^, ^16^, ^17^. *peg‐344*, *iucA*, *iroN*, *rmpA*, and *rmpA2* were usually found in virulence plasmids, *yptP* and *clbH* in ICE*Kp*, *mceG* in Tn*7399*, and *allS* in *all*_island (Figure 3).

Each hvKp isolate contained a virulence plasmid with at least one of *iucA* and *peg‐344*. These plasmids in the 2097 hvKp isolates were categorized into six Inc groups based on primary replicons: IncFIB‐4.2 (*n* = 1717), IncFIB‐4.1 (*n* = 200), IncHI3 (*n* = 159), IncFIB‐6.1 (*n* = 12), IncFIB‐8.1 (*n* = 7), and IncFIB‐7.1 (*n* = 2) (Table S2). Additionally, considering auxiliary and truncated nonfunctional Inc replicons, we further subdivided them into 32 Inc subgroups (Table S2). The top four Inc subgroups, namely, IncFIB‐4.2 + ΔIncHI3 (45.40%, 952/2097), IncFIB‐4.2 + IncHI3 (24.51%, 514/2097), IncFIB‐4.2 + IncFIB‐8.1 (10.40%, 218/2097), and IncHI3 + IncFIB‐6.1 (4.67%, 98/2097), were also mostly prevalent in the top six CGs (Figure S5A).

pSGH10 (CP025081), pK2044 (AP006726), p23‐1 (CP030321), and p721005‐1 (CP030293), which were selected as their reference plasmids, respectively, contained the following three conserved functional modules: specific replicons [IncFIB‐4.2 + ΔIncHI3: *repA*_IncFIB‐4.2_ and Δ*repHI3B*; IncFIB‐4.2 + IncHI3: *repA*_IncFIB‐4.2_ and *repHI3B*; IncFIB‐4.2 + IncFIB‐8.1: *repA*_IncFIB‐4.2_ and *repA*_IncFIB‐8.1_; IncHI3 + IncFIB‐6.1: *repHI3B* and *repA*_IncFIB‐6.1_], maintenance (*parAB*), and tellurium resistance (*ter*) (Figure S5B). In addition, the first three plasmids carried a full array of virulence genes *peg‐344*, *iuc*, *iro*, *rmpA/rmpA2*, and the *sil‐cop* region containing *sil* (silver resistance) and *cop* (copper resistance), but p721005‐1 lacked *peg‐344*, *iro*, *rmpA*, and the complete *sil‐cop* region (Figure S5B). Only p721005‐1 had a complete set of conjugation module, suggesting its potential self‐conjugative ability. In contrast, the other three plasmids displayed incomplete conjugation modules and thus were likely non‐self‐conjugative.

A significant proportion (77.40%) of the 2097 strains contained a chromosomal ICE*Kp*, harboring *yptP* or *yptP* + *clbH* (Tables S1 and S3). A total of 10 ICE*Kp* variants were identified, and the top four ICE*Kp* variants, namely, ICE*Kp10* (35.00%, 734/2097), ICE*Kp3* (30.00%, 629/2097), ICE*Kp12* (3.91%, 82/2097), and ICE*Kp1* (2.72%, 57/2097), were also most frequently found in the top six CGs (Table S3 and Figure S5A). Structurally, all these top four ICE*Kp* variants contained attachment sites (*attL/attR*) at both ends, one or two *int*, conjugal transfer regions, and *ybt*; in contrast, *clb* was only found in ICE*Kp10*, while the Zn^2+^/Mn^2+^ metabolism module was only presented in ICE*Kp10* and ICE*Kp12* (Figure S5C).

The chromosomal Tn*7399* and *all*_island displayed highly conserved modular structures, and their prevalence rates among the 2097 hvKp isolates were 29.38% and 32.24%, respectively (Table S3). Both Tn*7399* and *all*_island were exclusive to CG23 but absent in the other five top CGs (Figure S5A). Tn*7399* contained *attL*/*attR* at its both sides, *int*, and *mce* locus (Figure S5D). *all*_island carried *all* but lacked any integrases/transposases (Figure S5E).

### Classification of dominant CGs into specific clades co‐evolving with VDCs

**Figure 4:**
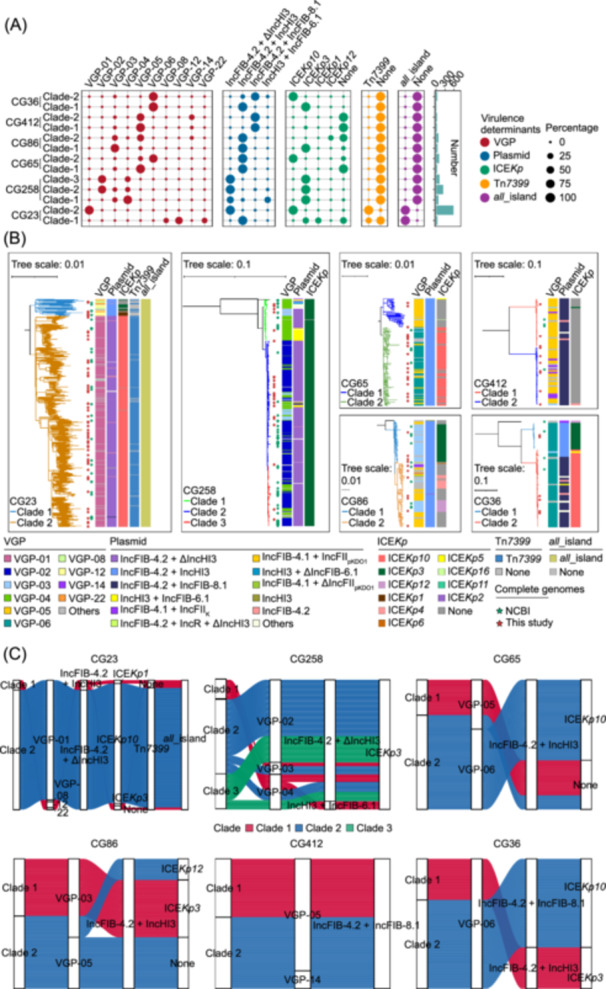
Correlation of VGPs and VAGEs within clades of the top six CGs. (A) Bubble charts showing the prevalence of the major 10 VGPs, four main types of virulence plasmids, four major ICE*Kp* variants, Tn*7399*, and *all*_island within the clades of the top six CGs, alongside a bar plot indicating the number of isolates per clade. Bubble size reflects prevalence degree. (B) Association of major VGPs and VAGEs across clades of the top six CGs. The six trees are the ML phylogenetic trees for the top six CGs (CG23, CG258, CG65, CG86, CG412, and CG36) based on their recombination‐free core SNPs (19,647, 17,936, 29,827, 26,537, 22,637, and 24,802) from 635, 430, 135, 128, 64, and 56 hvKp isolates, respectively. Green and red stars denote complete genomes from NCBI and sequenced in this study, respectively. (C) Alluvial diagrams showing major VDCs, including VGP, virulence plasmid, ICE*Kp*, Tn*7399*, and *all*_island, within clades of the top six CGs. Clades 1, 2, and 3 are colored red, blue, and green, respectively. VAGE, virulence‐associated genetic element; VDC, virulence determinant combination.

We then constructed six ML phylogenetic trees for the top six CGs (CG23, CG258, CG65, CG86, CG412, and CG36, respectively) based on their own recombination‐free core SNPs. CG258 was divided into three major clades, while each of the other five CGs formed two primary clades. Mapping VGPs and VAGEs onto these identified clades in the trees demonstrated the distinct VDC patterns among these CGs and their clades (Figure 4A,B).

CG23 represented the most prevalent and classical hvKp, and showed distinct VDC patterns between its two clades CG23‐Clade 1 (7.40%, 47/635) and CG23‐Clade 2 (92.60%, 588/635) (Figure 4B). On the one hand, CG23‐Clade 1 mainly contained VGP‐12 (53.19%), VGP‐22 (21.28%), and VGP‐08 (17.02%), while 29.79% and 31.91% harbored ICE*Kp1* and ICE*Kp3*, respectively, and 38.30% lacked any ICE*Kp*, showing high diversity in VGPs and ICE*Kp* variants in this clade. 27.66% carried Tn*7399,* while the rest had none (Figure 4A). In contrast, 95.74% harbored IncFIB‐4.2 + IncHI3 virulence plasmids, and all had *all*_island, indicating the high conservation of these two VAGEs in CG23‐Clade 1 (Figure 4A). Here, VGP‐12 – IncFIB‐4.2 + IncHI3–ICE*Kp1*–*all*_island was identified as the most prevalent VDC for CG23‐Clade 1, and only accounted for 29.79% of CG23‐Clade 1 isolates (Table S4A). On the other hand, 84.69% of CG23‐Clade 2 isolates corresponded to the VDC of VGP‐01–IncFIB‐4.2 + ΔIncHI3 – ICE*Kp10 *– Tn*7399*–*all*_island (Table S4A). In summary, CG23 showed a crucial evolutionary trajectory shift from the earlier, smaller, and more diverse CG23‐Clade 1 (with greater diversity in VDCs) to the more prevalent and highly clonal CG23‐Clade 2 (with high conservation in VDCs).

As the most typical CG of KPC‐encoding *K. pneumoniae* ^31^, CG258 was identified herein as the second most prevalent CG of hvKp. The three clades of CG258‐Clade 1 (17.91%, 77/430), CG258‐Clade 2 (55.12%, 237/430), and CG258‐Clade 3 (26.51%, 114/430) had different profiles of VDCs (Figure 4B and Table S4A). In CG258‐Clade 1, three primary VDCs were identified: VGP‐04–IncHI3 + IncFIB‐6.1–ICE*Kp3* (31.17%, *n* = 24), VGP‐04–IncFIB‐4.2 + ΔIncHI3–ICE*Kp3* (28.57%, *n* = 22), and VGP‐03–IncFIB‐4.2 + ΔIncHI3–ICE*Kp3* (14.29%, *n* = 11) (Table S4A). There were three major VDCs in CG258‐Clade 2: VGP‐02–IncFIB‐4.2 + ΔIncHI3–ICE*Kp3* (69.20%, *n* = 164), VGP‐04 – IncFIB‐4.2 + ΔIncHI3–ICE*Kp3* (12.24%, *n* = 29), and VGP‐03–IncFIB‐4.2 + ΔIncHI3–ICE*Kp3* (10.13%, *n* = 24) (Table S4A). Two predominant VDCs were found in CG258‐Clade 3: VGP‐02–IncFIB‐4.2 + ΔIncHI3–ICE*Kp3* (67.54%, *n* = 77), and VGP‐04–IncFIB‐4.2 + ΔIncHI3–ICE*Kp3* (23.68%, *n* = 27) (Table S4A). The above diverse co‐evolution patterns of VDCs for different CG258 primarily stemmed from the variety of VGPs. Additionally, VAGEs of CG258 demonstrated a degree of conservation: ICE*Kp* of almost all CG258 isolates was ICE*Kp3*, and all CG258 isolates lacked Tn*7399* and *all*_island (Figure 4A). Notably, over half of CG258 strains had the VDC of VGP‐02–IncFIB‐4.2 + ΔIncHI3–ICE*Kp3* (56.05%, 241/430) (Table S4B).

CG65 revealed two clades CG65‐Clade 1 (26.67%, 36/135) and CG65‐Clade 2 (73.33%, 99/135) with different VDCs (Figure 4B and Table S4A). CG65‐Clade 1 predominantly displayed the VDC of VGP‐05–IncFIB‐4.2 + IncHI3, accounting for 91.67% of this clade (Table S4A). CG65‐Clade 2 primarily harbored the VDC of VGP‐06–IncFIB‐4.2 + IncHI3–ICE*Kp10*, representing 75.76% of the clade (Table S4A). CG65‐Clade 1 lacked ICE*Kp,* whereas CG65‐Clade 2 acquired ICE*Kp10*, and this acquisition introduced *ybtP* and *clbH* in CG65‐Clade 2, marking a transition from VGP‐05 (CG65‐Clade 1) to VGP‐06 (CG65‐Clade 2) (Figure 4A).

For CG86, its two clades CG86‐Clade 1 (38.28%, 49/128) and CG86‐Clade 2 (60.94%, 78/128) also showed distinct VDCs (Figure 4B and Table S4A). 77.55% of CG86‐Clade 1 isolates showed the VDC of VGP‐03–IncFIB‐4.2 + IncHI3–ICE*Kp3* (Table S4A). CG86‐Clade 2 included two major VDCs, namely, VGP‐05–IncFIB‐4.2 + IncHI3 (43.59%) and VGP‐03–IncFIB‐4.2 + IncHI3–ICE*Kp12* (17.95%) (Table S4A). Compared to the first one of Clade 2 as the most prevalent VDC, the latter one acquired *ybtP*‐carrying ICE*Kp12*. Additionally, almost all CG86 isolates (98.44%) had IncFIB‐4.2 + IncHI3 virulence plasmids and all lacked Tn*7399* and *all*_island (Figure 4A).

CG412 (*n* = 64) displayed high conservation in VDC across its two clades, with the majority (44/64, 68.75%) showing the VDC of VGP‐05–IncFIB‐4.2 + IncFIB‐8.1 (Figure 4B and Table S4A). Remarkably, 96.88% of CG412 isolates lacked any ICE*Kp* variants, and all were devoid of Tn*7399* and *all*_island (Figure 4A).

CG36 (*n* = 56) comprised two clades CG36‐Clade 1 (*n* = 14) and CG36‐Clade 2 (*n* = 41) displaying distinct VDCs (Figure 4B). CG36‐Clade 1 was exclusively associated with VGP‐06–IncFIB‐4.2 + IncHI3–ICE*Kp3*, while CG36‐Clade 2 predominantly (73.17%) displayed VGP‐06–IncFIB‐4.2 + IncFIB‐8.1–ICE*Kp10* (Table S4A). None of the CG36 isolates contained Tn*7399* and *all*_island (Figure 4A).

Overall, for the top six CGs, we identified 17 major VDCs, which comprised 10 main VGPs (VGP‐01 to VGP‐06, VGP‐08, VGP‐12, VGP‐14, and VGP‐22), top four Inc groups of virulence plasmids (IncFIB‐4.2 + ΔIncHI3, IncFIB‐4.2 + IncHI3, IncFIB‐4.2 + IncFIB‐8.1, and IncHI3 + IncFIB‐6.1), top four ICE*Kp* variants (ICE*Kp10*, ICE*Kp3*, ICE*Kp1*, and ICE*Kp12*), and two additional conserved Tn*7399* and *all*_island (Figure 4C and Table S4B). Significantly, CG23‐Clades 1/2, CG258‐Clades 1/2/3, CG65‐Clades 1/2, CG86‐Clades 1/2, CG412‐Clades 1/2, and CG36‐Clades 1/2 harbored 4/1, 3/3/2, 1/2, 1/2, 1/2, and 1/1 distinct VDCs, respectively, indicating the strong co‐evolution of different VDCs with the distinct clades in the top six CGs (Figure 4A,C and Table S4A).

### Structural conservation and variation of VAGEs

We further analyzed the structural variations of the four VAGEs across the top six CGs and their clades using 187 complete genomes (comprising 102 sequenced in this study and 85 obtained from GenBank). The results revealed significant structural diversity in the same Inc groups of virulence plasmids or in the ICE*Kp* variants across different CGs/clades (Figure S6A–M). In contrast, Tn*7399* and *all*_island showed highly conserved structures (Figure S6N).

Plasmids of the most prevalent Inc group IncFIB‐4.2 + ΔIncHI3 were predominantly found in CG23‐Clade 2 and CG258‐Clades 1/2/3. Within CG23‐Clade 2, IncFIB‐4.2 + ΔIncHI3 plasmids maintained a sole structure identical to the reference plasmid pSGH10 (Figure S6B). In contrast, IncFIB‐4.2 + ΔIncHI3 plasmids in CG258 showed significant clade‐specific structural variations, marked by varying genomic deletions: (i) CG258‐Clade 1: ~56 kb deletion encompassing *peg‐344*, *rmpA*, and *iro* (Figure S6C); (ii) CG258‐Clade 2: two significant deletions, namely, ~15 kb deletion including partial *iroN* and complete *iroBCD*, and ~3 kb deletion containing five antioxidant genes^30^ (Figure S6D); and (iii) CG258‐Clade 3: two deletions identical to CG258‐Clade 2, and an additional ~26 kb deletion encompassing *sil*‐*cop* region (Figure S6E).

Plasmids of the second most prevalent Inc group IncFIB‐4.2 + IncHI3 were primarily located in CG23‐Clade 1, CG65‐Clades 1/2, CG86‐Clades 1/2, and CG36‐Clade 1. IncFIB‐4.2 + IncHI3 plasmids in CG23‐Clade 1 mirrored the structure of the reference plasmid pK2044, indicating high conservation (Figure S6A). Conversely, the plasmids in CG65‐Clades 1/2, CG86‐Clades 1/2, and CG36‐Clade 1 showed varying structural deletions: (i) CG65‐Clades 1/2, CG86‐Clades 1/2, and CG36‐Clade 1 harbored a common ~12 kb deletion, encompassing an ~3 kb segment linked to antioxidant capacity (Figure S6F–I,L). (ii) Specific deletions were noted in different clades: CG65‐Clades 1/2 had an ~2 kb deletion containing two genes of unknown functions (Figure S6F,G); CG86‐Clades 1/2 included an ~2 kb deletion with a gene encoding DNA polymerase III subunit epsilon and the other coding for a CRISPR‐associated protein (Figure S6H,I). CG36‐Clade 1 had a significant ~10 kb deletion upstream of *repHI3B* (Figure S6L).

Additionally, plasmids of the third most prevalent Inc group IncFIB‐4.2 + IncFIB‐8.1 were primarily found in CG412‐Clades 1/2 and CG36‐Clade 2. Relative to the reference plasmid p23‐1, IncFIB‐4.2 + IncFIB‐8.1 plasmids in CG412‐Clade 1 and CG36‐Clade 2 showed considerable structural conservation (Figure S6J,M), but those in CG412‐Clade 2 displayed varying deletions (Figure S6K). Furthermore, plasmids of the fourth most prevalent Inc group IncHI3 + IncFIB‐6.1 were predominantly identified in CG258‐Clade 1 (Figure S6C), and showed a largely conserved structure, underscoring the stability of IncHI3 + IncFIB‐6.1 plasmids within this clade.

The most prevalent ICE*Kp* variant ICE*Kp10* was predominantly observed in CG23‐Clade 2, CG65‐Clade 2, and CG36‐Clade 2. 23.21% of CG23‐Clade 2 isolates and 26.67% of CG65‐Clade 2 isolates showed deletions in the Zn^2+^/Mn^2+^ module of ICE*Kp10* (Figure S6B,G). Conversely, ICE*Kp10* counterparts in CG36‐Clade 2 isolates maintained a highly conserved structure like the reference ICE*Kp10* (Figure S6M). Furthermore, ICE*Kp3* was primarily found in CG23‐Clade 1, CG258‐Clades 1/2/3, CG86‐Clade 1, and CG36‐Clade 1. The structure of ICE*Kp3* in CG23‐Clade 1, CG86‐Clade 1, and CG36‐Clade 1 remained highly conserved compared to the reference ICE*Kp3* (Figure S6A,H,L). However, in CG258‐Clades 1/2/3, ICE*Kp3* counterparts showed two significant deletions: one approximately 3 kb within *virB3‐4*–*virB6* region and the other (~2 kb) at the 3′‐end including two genes of unknown functions (Figure S6C–E). Additionally, ICE*Kp1* was predominantly found in CG23‐Clade 1, showcasing a conserved structure similar to the reference ICE*Kp1* (Figure S6A). In contrast, ICE*Kp12*, primarily located in CG86‐Clade 2, was noted for the absence of the Zn^2+^/Mn^2+^ module (Figure S6I).

Overall, our analysis revealed that the same types of virulence plasmids or the ICE*Kp* variants showed considerable structural variations, primarily characterized by various extents of deletions, across different dominant CGs and their clades. These variations might arise from adaptive evolutionary processes tailored to each CG and clade, potentially enhancing the adaptability of hvKp isolates to human microenvironments and increasing their transmissibility among human individuals.

### Prevalence of MDR‐hvKp, CR‐hvKp, and hv‐CRKp

**Figure 5:**
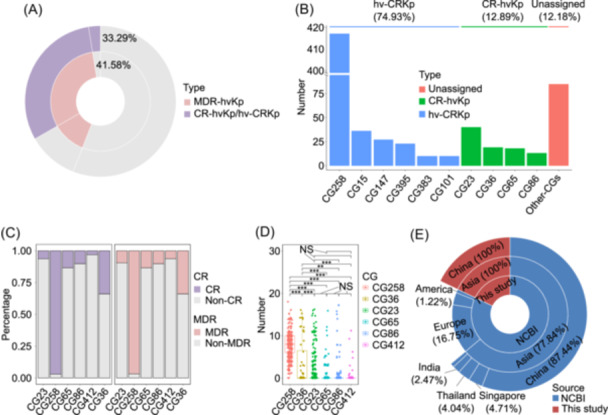
Severe AMR in hvKp isolates. (A) Distribution of MDR‐hvKp and CR‐hvKp/hv‐CRKp isolates among 2097 hvKp isolates. (B) Prevalence of hv‐CRKp and CR‐hvKp among 698 carbapenem‐resistant hvKp isolates. “Unassigned” refers to isolates ambiguously classified as hv‐CRKp or CR‐hvKp. (C) Prevalence of CR/non‐CR and MDR/non‐MDR hvKp isolates within the top CGs; the *y*‐axis indicates percentages. (D) Box‐scatter plot showing the number of resistance genes in hvKp isolates from the top six CGs. *p* values were calculated using a *t*‐test (*p < 0.05; **p < 0.01; ***p < 0.001; NS, not significant). (E) Geographic distribution of the 698 CR‐hvKp/hv‐CRKp isolates. AMR, antimicrobial resistance; CR, carbapenem‐resistant; MDR, multidrug‐resistant.

We analyzed the distribution of known AMR genes in the 2097 hvKp isolates, identifying a total of 198 AMR genes belonging to 11 categories such as resistance to carbapenems, aminoglycosides, and quinolones (Table S1). Among these, four AMR genes *bla*_SHV_, *fosA*, *oqxA*, and *oqxB* demonstrated >80.00% prevalence rates (Table S1). Our findings revealed the significant prevalence rates of MDR‐hvKp and CR‐hvKp/hv‐CRKp in the 2097 hvKp isolates: 41.58% (*n* = 872) were classified as MDR‐hvKp and 33.29% (*n* = 698) as CR‐hvKp/hv‐CRKp (Figure 5A). Furthermore, among the 698 CR‐hvKp/hv‐CRKp, 74.93% (523/698) were identified as hv‐CRKp, 12.89% (90/698) as CR‐hvKp, and 12.18% (85/698) were ambiguously classified as hv‐CRKp or CR‐hvKp (Figure 5B). The above data indicate the severe AMR in hvKp.

The 523 hv‐CRKp strains corresponded to CG258, CG15, CG147, CG395, CG383, and CG101, and notably, CG258 accounted for 79.73% (417/523) and thus represented the most prevalent hv‐CRKp CG (Figure 5B). The 90 CR‐hvKp strains primarily consisted of CG23, followed by CG36, CG65, and CG86 (Figure 5B). This indicates that hv‐CRKp was more prevalent and suitable for survival in hospital settings than CR‐hvKp, consistent with the previous study^27^.

As expected, almost all CG258 isolates belonged to MDR‐hvKp (96.74%, 416/430) and hv‐CRKp (96.98%, 417/430) (Figure 5C and Table S5), which was known as the most popular CG of CRKp as well as hv‐CRKp worldwide^23^, ^27^. CG258 displayed an average of eight AMR genes per isolate, which was significantly higher than all the other five major CGs (Figure 5D). CG36 also showed relatively high ratios of MDR‐hvKp (33.93%, 19/56) and CR‐hvKp (33.93%, 19/56), with an average of four AMR genes per isolate (Figure 5C,D). In contrast, the remaining four CGs showed lower ratios (<15.00%) of MDR‐hvKp and CR‐hvKp/hv‐CRKp, with fewer AMR genes per isolate (Figure 5C,D). Moreover, among the 28 major CGs (each *n* > 10), 13 displayed an MDR‐hvKp proportion >50.00%, including CG258, CG15, CG25, CG147, CG395, CG37, CG700, CG43, CG35, CG2096, CG101, CG383, and CG48; eight CGs had a CR‐hvKp/hv‐CRKp proportion >50.00%, including CG258, CG15, CG147, CG395, CG2096, CG101, CG383, and CG48 (Table S5). These findings underscore the urgent need for close monitoring and targeted interventions for these high‐risk CGs.

Our study revealed that 81.81% (571/698) of the CR‐hvKp/hv‐CRKp isolates were isolated from Asia (Figure 5E). After excluding our self‐collected 900 hvKp isolates to avoid data collection bias, even in the GenBank dataset, the proportion of CR‐hvKp/hv‐CRKp from Asia still reached 77.84% (446/573), with 87.44% (390/446) collected from China (Figure 5E). Several studies also highlighted that CR‐hvKp/hv‐CRKp was predominantly reported in Asia, particularly in China^6^, ^24^, ^32^. Therefore, there is an urgent need for Asian countries, especially China, to implement effective strategies to control the spread of CR‐hvKp/hv‐CRKp.

Additionally, our analysis revealed significant differences between CR‐ and carbapenem‐sensitive (CS)‐ hvKp isolates in terms of the CGs, VGPs, VAGEs, and VDCs. Specifically, CR‐hvKp were primarily assigned to CG258 and CG15, while CS‐hvKp were predominantly assigned to CG23, CG65, CG86, and CG412. Regarding VGPs, CR‐hvKp mainly harbored VGP‐02/03/04, while CS‐hvKp primarily carried VGP‐01/05/06. For VAGEs, both CR‐ and CS‐hvKp shared the predominant IncFIB‐4.2 + ΔIncHI3 virulence plasmid. However, ICE*Kp3* and ICE*Kp12* were mainly observed in CR‐hvKp, whereas ICE*Kp10* was primarily found in CS‐hvKp. The majority of CR‐hvKp lacked both Tn*7399* and *all*_island, while at least 33.81% of CS‐hvKp harbored them. Finally, for VDCs, CR‐hvKp isolates included VDCs of VGP‐02–IncFIB‐4.2 + ΔIncHI3–ICE*Kp3*, VGP‐04–IncFIB‐4.2 + ΔIncHI3–ICE*Kp3*, VGP‐03–IncFIB‐4.2 + ΔIncHI3–ICE*Kp3*, VGP‐04–IncHI3 + IncFIB‐6.1–ICE*Kp3*, and VGP‐04–IncFIB‐4.2 + IncHI3–ICE*Kp12*. Meanwhile, the CS‐hvKp contained VDCs of VGP‐01–IncFIB‐4.2 + ΔIncHI3–ICE*Kp10*–Tn*7399*–*all*_island, VGP‐06–IncFIB‐4.2 + IncHI3–ICE*Kp10*, VGP‐05–IncFIB‐4.2 + IncHI3, VGP‐05–IncFIB‐4.2 + IncFIB‐8.1, and VGP‐05–IncFIB‐4.2 + IncHI3 (Table S6).

### Phylogeny and evolutionary history of CG23 and CG258

**Figure 6:**
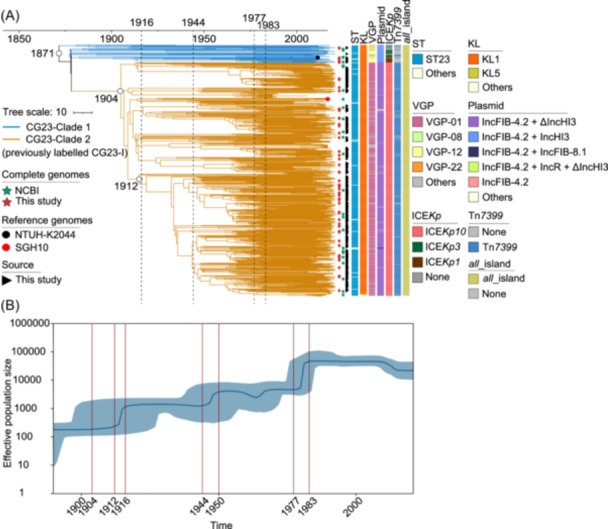
Evolutionary history of CG23 hvKp. (A) Time‐calibrated MCC Bayesian phylogeny based on 19,647 recombination‐free core SNPs from 600 nonredundant CG23 hvKp isolates, subsampled from an initial 635 ones. Distinct clades are indicated by colored clusters. Green and red stars denote complete genomes from NCBI and sequenced in this study. Black and red circles show hvKp reference genomes NTUH‐K2044 and SGH10, respectively. Black triangles indicate hvKp isolates sequenced in this study. (B) Bayesian skyline plot showing the effective population size of the 600 CG23 hvKp isolates over time. The shaded area represents the 95% highest posterior density interval. MCC, maximum clade credibility; SNPs, single‐nucleotide polymorphisms.

We conducted phylogeny and evolutionary history analyses on two key CGs: CG23 (the most prevalent hvKp CG) and CG258 (the most prevalent hv‐CRKp CG). Our sequencing of the nonredundant 600 CG23 isolates led to the identification of 19,647 recombination‐free SNPs, and accordingly, we constructed a Bayesian maximum clade credibility (MCC) tree, which was time‐calibrated for historical context (Figure 6A). This analysis divided CG23 into two primary clades: the earlier and minor CG23‐Clade 1, and the recent and overwhelming CG23‐Clade 2 (previously referred to as CG23‐I in Holt's paper^21^, comprising 93.00% of the 600 CG23 isolates) (Figure 6A). This distribution was in agreement with findings from the above ML tree. The most recent common ancestors (MRCAs) of CG23 were traced back to the year of 1871 (Figure 6A). Notably, Bayesian skyline plots indicated the estimated emergence of CG23‐Clade 2 around the year of 1904, corroborating Holt's findings^21^. Subsequent significant population expansions were observed in the periods around 1912 to 1916, 1944 to 1950, and 1977 to 1983, indicating periods of significant outbreaks (Figure 6B). The 1912 expansion notably corresponded to the rise of the largest subclade within CG23‐Clade 2 (Figure 6A). Additionally, we also performed Bayesian skyline plot analysis of isolates with the most prevalent VDC (VGP‐01–IncFIB‐4.2 + ΔIncHI3–ICE*Kp10*–Tn*7399*–*all*_island) within CG23. The result further confirmed the continuous and robust expansion of these isolates (Figure S7), underscoring their potential to become even more dominant in the future. Therefore, it was crucial to closely monitor the spread of these CG23 isolates and develop effective strategies to manage and mitigate the associated risks.

**Figure 7:**
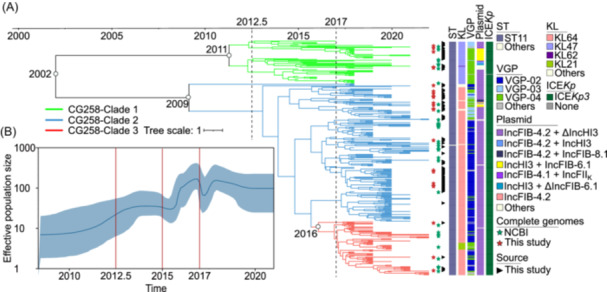
Evolutionary history of CG258 hvKp. (A) Time‐calibrated MCC Bayesian phylogeny based on 17,936 recombination‐free core SNPs from 319 nonredundant CG258 isolates, subsampled from an initial 430 ones. Distinct clades are shown as colored clusters. Green and red stars indicate complete genomes from NCBI and sequenced in this study. Black triangles denote hvKp isolates sequenced in this study. (B) Bayesian skyline plot showing the effective population size of the 319 CG258 isolates over time. The shaded area represents the 95% highest posterior density interval.

For CG258, we analyzed nonredundant 319 unique isolates and constructed a time‐calibrated Bayesian MCC tree based on 17,936 recombination‐free SNPs. This analysis identified three clades: CG258‐Clade 1, CG258‐Clade 2, and CG258‐Clade 3, which was also consistent with the above ML tree (Figure 7A). The MRCAs for these clades were dated to 2002, with specific dates for each clade being 2011, 2009, and 2016, respectively. Bayesian skyline analysis revealed that the CG258 population began expanding in 2012, with a significant surge after 2015 (Figure 7B). However, post‐2017, the Bayesian skyline plot showed some irregular fluctuations, possibly due to sampling time biases (Figure 7B).

Overall, our findings highlighted the intricate evolutionary trajectories of CG23 and CG258. The consistency of the clades of each CG between Bayesian MCC and ML trees confirmed their identical co‐evolutionary patterns. Understanding these patterns was vital for developing effective countermeasures against these two critical hvKp CGs.

### Assessment of hypermucoviscosity and virulence phenotypes

**Figure 8:**
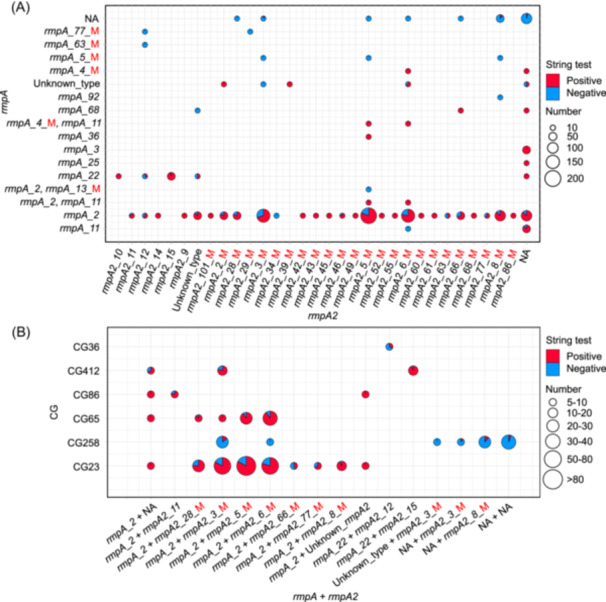
Correlation between *rmpA* and *rmpA2* subtypes and the hypermucoviscosity phenotype assessed by the string test. (A) Correlation between 67 combinations of *rmpA* and *rmpA2* subtypes and the hypermucoviscosity phenotype. *rmpA* subtypes are shown on the vertical axis and *rmpA2* subtypes are shown on the horizontal axis. (B) Correlation between *rmpA *+ *rmpA2* subtype combinations and the hypermucoviscosity phenotype in the top six CGs. Only combinations with ≥5 strains per CG are shown. The size of each circle indicates the number of hvKp isolates; red and blue represent positive and negative string test results, respectively. M indicates mutations leading to amino acid sequence deletions.

The hypermucoviscosity and virulence phenotypes of hvKp isolates were evaluated using the string test and the *Galleria mellonella* infection model, respectively. Initially, 629 (69.89%) of all our 900 sequenced isolates were positive for hypermucoviscosity (Table S7). In addition, among the nine key virulence genes, *rmpA* and *rmpA2* were known to directly link to the hypermucoviscosity phenotype^7^. We categorized the subtypes of these two genes within each of the 900 hvKp isolates. Among the 900 hvKp isolates, eight subtype combinations of *rmpA* + *rmpA2* (each *n* ≥ 10) showed a string test positivity rate ≥80.00% (Figure 8A). These combinations included *rmpA_2* alone (86.79%), *rmpA_2* + *rmpA2_2* (81.82%), *rmpA_2* + *rmpA2_5* (80.98%), *rmpA_2* + *rmpA2_8* (86.67%), *rmpA_2* + *rmpA2_28* (80.49%), *rmpA_3* alone (100.00%), *rmpA_11* alone (87.50%), and *rmpA_22* + *rmpA2_15* (95.24%). Remarkably, all 16 strains carrying only *rmpA_3* displayed a 100.00% positivity rate (Figure 8A). Hence, strains that show these gene combinations (particularly those with the *rmpA_3* subtype) should be closely monitored due to their high virulence potential.

We further explored the correlation between hypermucoviscosity genotypes and phenotypes across 17 CGs (each *n* ≥ 10). Among these, 14 CGs had positivity rates ≥50.00%. Notably, within the top six CGs, CG23, CG65, CG86, and CG412 showed positivity rates >80.00%, while CG258 and CG36 had rates of 8.20% and 47.83%, respectively (Table S7).

Of the 527 isolates from CG23, CG65, CG86, and CG412, 83.49% (440/527) were string test‐positive. Remarkably, 99.09% (436/440) carried at least one functional *rmpA*/*rmpA2* gene, including 29 with both functional *rmpA* and *rmpA2* genes, 21 with a functional *rmpA* and an unknown type of *rmpA2*, 28 with only a functional *rmpA*, and 358 with a functional *rmpA* and a mutated *rmpA2* (Table S8). Mutations within *rmpA2* of the 358 isolates resulted in deletions of the amino acid sequences, likely rendering these genes nonfunctional^29^, ^33^. Consequently, 386 isolates with only a functional *rmpA* showed a hypermucoviscosity phenotype, 97.15% (375/386) of which had the *rmpA_2* subtype, highlighting the critical role of *rmpA*, particularly *rmpA_2*, in hypermucoviscosity phenotype regulation (Figure 8B and Table S8). However, a minority of isolates with a functional *rmpA_2* alone, or even both functional *rmpA* and *rmpA2*, were string test‐negative (Figure 8B), suggesting that other factors might also contribute to the hypermucoviscosity phenotype. In CG258, 112 string test‐negative isolates included 37 lacking both *rmpA* and *rmpA2*, 38 lacking *rmpA* but with a mutated *rmpA2*, and one with both mutated genes. Additionally, 30 isolates with a functional *rmpA* (*rmpA_2*) and a mutated *rmpA2* (*rmpA2_3* or *rmpA2_6*) showed a non‐hypermucoviscosity phenotype (Figure 8B and Table S8). In CG36, the common “*rmpA_22* + *rmpA2_12*” (both functional) combination had a 33.33% positivity rate (Figure 8B).

Using the *G. mellonella* infection model, we further selected 162 representative isolates, including 117 CG23 isolates and 45 CG258 isolates, to assess differences in their virulence across CGs and their respective clades. The virulence of CG23 isolates was significantly higher than that of CG258 isolates (Figure S8A). Within CG23, isolates of the more prevalent CG23‐Clade 2 showed significantly lower virulence compared to those of the earlier CG23‐Clade 1 (Figure S8B). Notably, nearly all CG23‐Clade 2 isolates (93.69%, 104/111) harbored the most prevalent VDC of the VGP‐01–IncFIB‐4.2 + ΔIncHI3–ICE*Kp10*–Tn*7399*–*all*_island (Figure S8C), indicating that these isolates might demonstrate an optimal balance between dissemination and virulence. Additionally, within CG258, isolates of the more prevalent CG258‐Clades 2 + 3 demonstrated markedly higher virulence than CG258‐Clade 1 isolates (Figure S8D). Notably, the differences of VDCs between CG258‐Clade 1 and CG258‐Clades 2 + 3 were primarily associated with VGPs and virulence plasmids, with almost all CG258 isolates containing ICE*Kp3*, and lacking Tn*7399* and *all*_island. Furthermore, the mainstream VDC of VGP‐04–IncHI3 + IncFIB‐6.1 in CG258‐Clade 1 showed significantly lower virulence than VGPs‐02/03/04–IncFIB‐4.2 + ΔIncHI3 in CG258‐Clades 2 + 3 (Figure S8E).

## DISCUSSION

**Figure 9:**
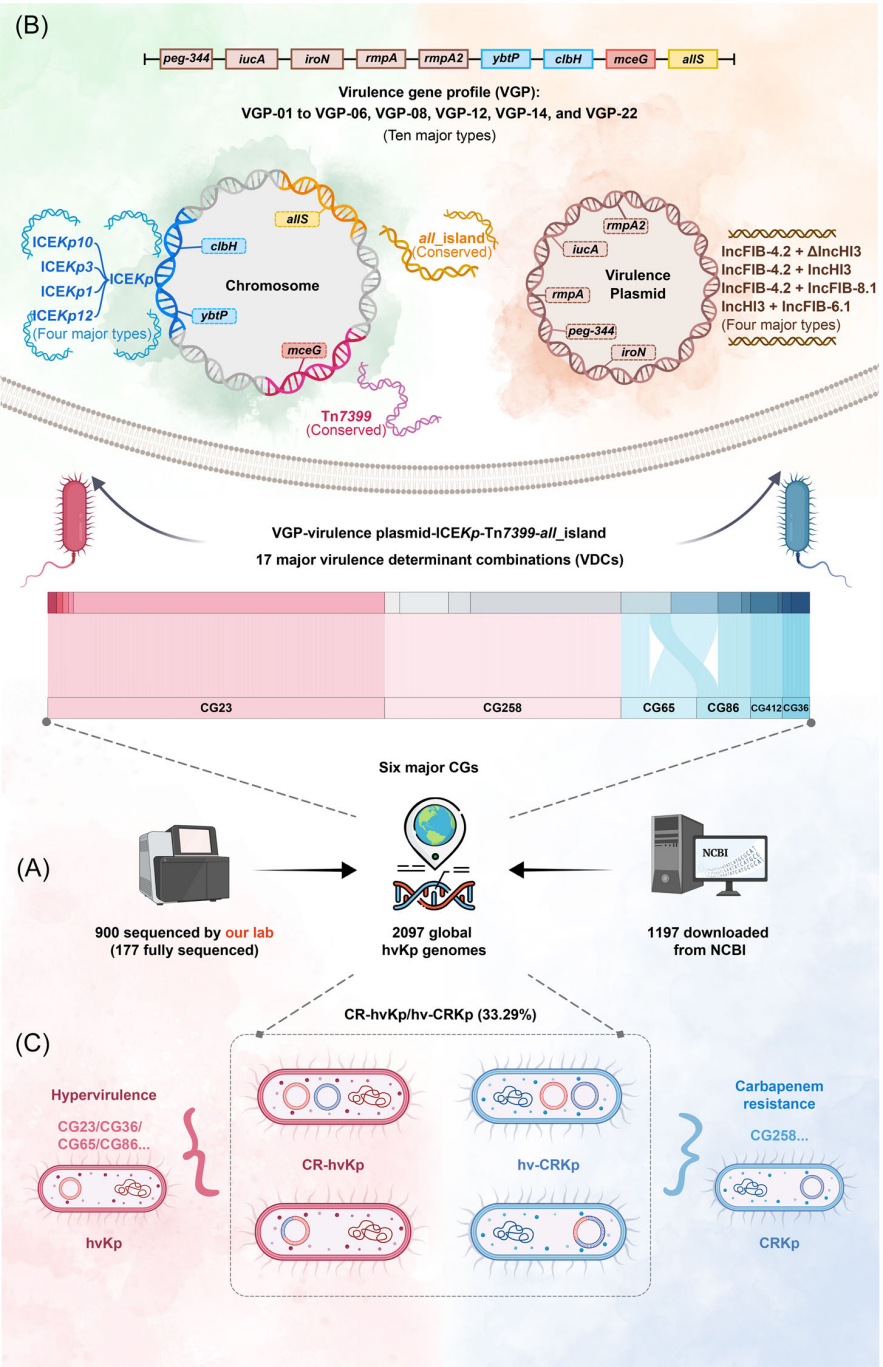
Schematic illustration of the global genomic landscape and evolutionary dynamics of hvKp. (A) Collection of 2097 global hvKp isolates. We collected a total of 2097 hvKp genomes, comprising 900 isolates sequenced (Illumina platform) in this study (including 177 fully sequenced using PacBio) and 1197 publicly available genomes from NCBI. (B) Strong genomic association and co‐evolution between CGs and VDCs. Among the isolates, six dominant CGs showed distinct advantageous VDCs, reflecting a strong genomic association and co‐evolution. These 17 major VDCs were linked to 10 VGPs, four virulence plasmid types, four ICE*Kp* variants, Tn*7399*, and *all*_island. (C) Two evolutionary pathways to convergence of hypervirulence and carbapenem resistance. Of the 2097 hvKp isolates, 33.3% were CR‐hvKp/hv‐CRKp, highlighting a serious public health threat. The convergence of hypervirulence and carbapenem resistance primarily follows two evolutionary routes: hvKp acquiring resistance plasmids or modules (blue) to evolve into CR‐hvKp, and CRKp acquiring virulence plasmids or modules (red) to evolve into hv‐CRKp.

Based on the largest global dataset of 2097 hvKp isolates to date, our study will provide the most comprehensive genomic epidemiological description of hvKp compared to previous studies^21^, ^30^. We thoroughly illustrated the classification and relationships among major CGs, VGPs, VAGEs, VDCs, and CR‐hvKp/hv‐CRKp, offering a systematic genomic landscape of global hvKp isolates (Figure 9). Despite the complex semi‐clonal population structure of hvKp, a trend toward the dissemination of multiple clonal complexes has become increasingly evident^2^, ^31^. Our study showed that the top six CGs account for 69.05% of the 2097 hvKp isolates, with CG23 and CG258 being the most predominant ones (30.28% and 20.51%, respectively) (Figure 2A). This clonal dissemination trend poses a substantial public health threat that warrants close attention.

What were the genetic bases behind the global spread of the top six CGs and their 13 major clades? The 17 major VDCs were highly correlated with different CGs and their clades, highlighting the strong co‐evolution of different VDCs with corresponding clades in dominant CGs (Figure 4C). Notably, the most prevalent VDC of VGP‐01–IncFIB‐4.2 + ΔIncHI3–ICE*Kp10*–Tn*7399*–*all*_island accounted for approximately 34.39% (498/1448) of the strains from the top six CGs, and it showed a strong correlation and co‐evolution with CG23‐Clade 2 (Table S4B). This VDC had all nine key virulence genes and the entire four VAGEs, and conferred significant infection and colonization advantages in the hosts, thereby making CG23‐Clade 2 the most prevalent and evolutionarily successful clade of hvKp worldwide. This was evident in the following three aspects. First, this VDC harbored a complete set of five known key virulence genes *peg‐344*, *iucA*, *iroN*, *rmpA*, and *rmpA2* on the virulence plasmids, and all of them had been proven to play crucial roles in the pathogenicity of hvKp^15^, ^16^, ^17^. Second, it contained ICE*Kp10*. Unlike the other three major ICE*Kp* variants ICE*Kp3*, ICE*Kp1*, and ICE*Kp12,* which only had the *ybt* locus, ICE*Kp10* contained both *ybt* and *clb*. The addition of *clb* promoted gut colonization and metastatic infection of hvKp^21^. Third, this VDC harbored additionally Tn*7399* and *all*_island, while almost all other VDCs had neither or only *all*_island. Microcin encoded by Tn*7399* endowed a competitive advantage for colonization in the colonic environment^2^, while the Δ*allS* mutant showed a significant reduction in virulence in vivo models^9^.

Previous studies indicated that most hvKp strains were commonly sensitive to antibiotics, whereas MDR‐Kp, especially CRKp, generally showed lower virulence^34^, ^35^, ^36^. However, in recent years, *K. pneumonia* strains with simultaneous hypervirulence and carbapenem resistance (true superbugs) have emerged and rapidly disseminated, causing difficult‐to‐treat invasive infections^30^, ^37^, ^38^, ^39^, and they could be categorized into two groups, namely, CR‐hvKp and hv‐CRKp, based on their distinct evolutionary paths^23^, ^24^. This study as well as the previous report^27^ indicated that hv‐CRKp was significantly more prevalent and better suited for survival in hospital settings than CR‐hvKp (Figure 5B). Among all hv‐CRKp CGs, CG258 accounted for an overwhelming proportion^24^, ^27^, ^32^ (Figure 5B). Alarmingly, our analysis showed that 33.29% were CR‐hvKp/hv‐CRKp (Figure 5A). Jiang et al.'s study^27^ also demonstrated that about 44.00% of global hvKp isolates showed both hypervirulence and carbapenem resistance. This staggering proportion underscores the urgent need for increased vigilance and monitoring of these isolates. Furthermore, previous studies^6^, ^24^, ^32^ as well as this study highlighted that CR‐hvKp/hv‐CRKp was predominantly reported in Asia, particularly in China (Figure 5E). There is an urgent need for Asian countries, especially China, to develop and implement effective strategies to control the spread of CR‐hvKp/hv‐CRKp.

CG258, originally characterized by CRKp^37^, ^40^, ^41^, had evolved into hv‐CRKp through the acquisition of a pK2044‐like virulence plasmid, resulting in increased virulence and a higher mortality rate in clinical infections^30^, ^42^. Our results revealed that 86.51% of the virulence plasmids in CG258 hv‐CRKp were assigned to IncFIB‐4.2 + ΔIncHI3 (Figure 4A). The following three primary factors might promote the prevalence of IncFIB‐4.2 + ΔIncHI3 virulence plasmids in CG258 hv‐CRKp. First, the high prevalence of KPC‐encoding plasmids in CG258 isolates could facilitate the integration of virulence plasmids, as evidenced by our study showing that 96.51% of CG258 hv‐CRKp strains contained *bla*_KPC_‐carrying plasmids (Table S1). Classical virulence plasmids such as pK2044 and pSGH10 were non‐self‐transmissible due to the lack of conjugation transfer modules^43^, ^44^. However, *bla*_KPC_‐carrying plasmids could mobilize non‐conjugative virulence plasmids from hvKp to CRKp^27^, ^44^, significantly promoting the integration and transmission of virulence plasmids into CG258 isolates. Second, CG258 hv‐CRKp showed a series of adaptive structural deletions to reduce the fitness cost of virulence plasmids, further facilitating its widespread dissemination^30^, ^45^. IncFIB‐4.2 + ΔIncHI3 plasmids in the more prevalent CG258‐Clades 2/3 showed three significant structural deletions: (i) an ~15 kb deletion in CG258‐Clades 2/3 (Figure S6D,E); (ii) an ~3 kb deletion in CG258‐Clades 2/3 (Figure S6D,E); and (iii) an ~26 kb deletion in CG258‐Clade 3 (Figure S6E). The ~15 kb deletion, which included partial *iroN* and the complete *iroBCD*, might contribute to the reduced virulence observed in these isolates compared to the classical CG23 hvKp^30^, ^46^. Notably, it was demonstrated that the above ~3 kb deletion regulated methionine metabolism, enhanced antioxidant capacity, and improved survival in macrophages, conferring CG258 hv‐CRKp with an evolutionary advantage^30^. In addition, the deletion of the ~26 kb *sil‐cop* region likely decreased the fitness cost of CG258‐Clade 3 isolates without the pressure of silver and copper. We thus reasoned to infer that these deletions might promote the dissemination of CG258 hv‐CRKp isolates by affecting specific metabolic pathways and providing evolutionary advantages. However, these mechanisms still need to be explored further. Third, classic CG258 CRKp was an important nosocomial pathogen with original resistance to many antimicrobials especially carbapenems, while the new integration of hypervirulent traits into CG258 hv‐CRKp would significantly facilitate its survival and dissemination in hospital settings.

Despite the relatively low proportion (12.89%) of CR‐hvKp in hvKp of CG23, CG36, CG65, and CG86 (Figure 5B), it was crucial to closely monitor CR‐hvKp. On the one hand, some of these CR‐hvKp strains evolved to acquire KPC‐encoding plasmids and thereby showed simultaneous hypervirulence and carbapenem resistance, and they might become increasingly prevalent through adaptive evolution in the future. On the other hand, the newly acquired resistance plasmid in CR‐hvKp could facilitate the horizontal transfer of nonconjugative virulence plasmids to other strains particularly CRKp strains, further promoting the formation and transmission of hv‐CRKp^27^.

Additionally, our string test results showed a low positive rate (8.20%) for CG258 hvKp isolates (Table S7), consistent with previous reports^47^. Also, the *G. mellonella* infection model revealed that the overall virulence of CG258 hvKp strains was significantly lower than that of typical CG23 hvKp strains (Figure S8). This aligned with previous studies^30^, ^46^, which confirmed that while CRKp‐CG258 strains showed increased virulence after acquiring virulence plasmids, their virulence remained lower than that of classic hvKp strains such as CG23.

Several factors might explain the relatively low proportion of hypermucoviscous strains among CG258 hvKp isolates. After acquiring virulence plasmids, these strains likely underwent adaptive changes under the pressures of high virulence and resistance, such as the loss or mutation of key virulence genes^30^, ^46^. Notably, the absence or mutation of both *rmpA* and *rmpA2* genes was a primary cause of the non‐hypermucoviscous phenotype in CG258 hvKp strains^33^. Furthermore, the lower virulence of CG258 strains compared to CG23 might be due to the loss or truncation of several other critical genes. Specifically, 56.74% of CG258 hvKp isolates carried truncated *iroN* and 29.53% lacked *peg‐344*, *iroN*, and *rmpA* (Figure 4). All CG258 hvKp isolates lacked *mceG* and *allS*, and 99.77% were deficient in *clbH*. These genetic alterations likely contributed to the lower virulence of CG258 hvKp strains. Additionally, we hypothesized that the nonhypermucoviscous phenotype in these strains represented an adaptive strategy to balance both high virulence and resistance^27^, ^48^.

In summary, we performed a comprehensive genomic epidemiological analysis of 2097 global hvKp isolates, including 900 newly sequenced in this study. We identified six major CGs, notably CG23 and CG258, and 17 major VDCs comprising 10 VGPs, four virulence plasmid types, four ICE*Kp* variants, Tn*7399*, and *all*_island. Each dominant CG showed distinct VDC patterns, reflecting strong genomic association and co‐evolution that contributed to their emergence and global dissemination. Notably, 33.29% of isolates were CR‐hvKp/hv‐CRKp, underscoring the serious threats of antimicrobial resistance. Among them, hv‐CRKp, particularly CG258, showed higher prevalence and adaptation in hospital settings due to intrinsic resistance and acquisition of virulence plasmids, highlighting the urgent need for targeted interventions. Overall, this study provides a comprehensive genomic landscape of hvKp (covering CGs/STs, VGPs, VAGEs, VDCs, AMR genes, and MDR and CR hvKp strains), offering valuable insights into its global dissemination and informing precise prevention and control strategies.

## MATERIALS AND METHODS

### Collection and screening of 2097 global hvKp isolates from 1932 to 2021

A total of 2097 hvKp isolates from 1932 to 2021 were screened and selected for this study, comprising 900 hvKp isolates that we sequenced using high‐throughput sequencing (177 of which were subjected to complete genome sequencing), and 1197 eligible hvKp genome sequences obtained from GenBank. The screening criteria for hvKp required two conditions: first, the average nucleotide identity (ANI) value greater than 95% when compared to the *K. pneumoniae* reference strain NJST258_1 (CP006928.1) to confirm the strain as Kp and second, the presence of at least one of two principal virulence genes (*peg‐344* and *iucA*)^19^ located on the virulence plasmid to confirm the strain as hvKp.

The detailed screening process for the 900 hvKp isolates we sequenced was as follows: we first collected 3000 clinical *K. pneumoniae* isolates from 46 hospitals across 15 provinces in China between 2004 and 2019. After excluding 52 unsuccessfully cultured isolates, we performed PCR to detect the *K. pneumoniae*‐specific *khe* gene^49^ and the two key virulence genes (*peg‐344* and *iucA*). Of the 2948 successfully tested isolates, 153 lacking *khe* and 1769 that did not contain either *peg‐344* or *iucA* were excluded. Genomic DNA from the remaining 1026 isolates was extracted using the Qiagen UltraClean Microbial DNA Isolation Kit (Catalog No. 12224‐50; Qiagen) and sequenced using the Illumina platform. After excluding 35 low‐quality sequencing samples and 91 isolates lacking virulence plasmids with either *peg‐344* or *iucA*, a total of 900 hvKp isolates were selected for further analysis (Figure S1 and Table S1).

From these 900 isolates, we further selected 177 representative hvKp strains for PacBio single‐molecule sequencing to obtain complete genomes (Table S1). The selection was based on the major VDCs within each CG (*n* ≥ 10) of the 900 hvKp isolates.

The screening process for the 1197 publicly available hvKp isolates from GenBank (spanning 1932 to 2021) was as follows: we applied the same hvKp screening criteria to 12,459 assembled *K. pneumoniae* genomes downloaded from GenBank (last accessed Dec 31, 2021). After removing 102 low‐quality samples, species identification was performed on the remaining 12,357 isolates using ANI values, which led to the exclusion of 151 non‐*K. pneumoniae* isolates. Additionally, 10,645 isolates that lacked both *peg‐344* and *iucA*, as well as 364 isolates without virulence plasmids containing at least one of these two genes, were excluded. Ultimately, 1197 hvKp isolates were selected for further analysis (Figure S1 and Table S1).

In total, in this study, 2097 hvKp isolates were analyzed, consisting of 900 hvKp isolates sequenced in‐house and 1197 publicly available hvKp isolates from GenBank.

### Genome sequencing, assembly, and quality control

The 900 hvKp isolates were sequenced using the Illumina HiSeq. 2000 platform^50^. Paired‐end libraries (with an average insert size of 350 bp) were constructed using the NEB Next Ultra DNA Library Preparation Kit (catalog No. E7370L; New England Biolabs) according to the manufacturer's recommended workflow^51^. Adapters and low‐quality reads were removed using FASTX‐Toolkit (http://hannonlab.cshl.edu/fastx_toolkit/), and high‐quality reads were assembled with SPAdes v3.9.0^52^ using the following parameters: ‐k 21, 33, 55 ‐‐careful ‐‐only‐assembler ‐‐cov‐cutoff “auto” data included in the final analysis had to meet the following criteria^41^: (1) genome size between 5–7 M; (2) ANI value greater than 95% when compared to the *K. pneumoniae* reference strain NJST258_1 (CP006928.1), as calculated by Pyani‐0.2.7^53^; and (3) over 80% of the genome positions had a coverage depth greater than 10×, determined by mapping raw reads to the NJST258_1 genome using Bowtie2^54^, with depth calculated by samtools depth v0.1.19^55^.

The 177 complete hvKp genome sequences were obtained using a PacBio Revio sequencer (Pacific Biosciences)^56^. The library (with an average size of 15 kb) was prepared using sheared genomic DNA (>2 µg) through SMRTbell® prep kit 3.0 (PacBio Ref. No. 102‐141‐700; Pacific Biosciences) according to the manufacturer's instructions. De novo sequence assembly was performed using hifiasm‐meta v0.13^57^ and Flye 2.9.2^58^, with manual circularization of chromosomal or plasmid sequences. Pilon v1.24^59^ was used to polish complete genome sequences with Illumina reads.

### MLST and KL typing

*SRST2* ^60^ was used to identify the ST of each isolate by mapping its Illumina sequencing reads to the Pasteur *Klebsiella* MLST Database (http://bigsdb.pasteur.fr/klebsiella/klebsiella.html). The STs of the assembled genomes were also identified through mlst (https://github.com/tseemann/mlst). All STs in the *Klebsiella* MLST database (last accessed December 14, 2021) were assigned to different CGs using goeBURST (http://www.phyloviz.net/goeburst/). KL types were identified using Kaptive (https://github.com/klebgenomics/Kaptive).

### Identification of virulence and AMR genes

The principal virulence genes (*peg‐344* and *iucA*) were screened in each sequenced hvKp isolate by PCR^21^, followed by amplicon sequencing on an ABI 3730 Sequencer. Virulence genes were identified through BLAST, using the Pasteur Institute's database (http://bigsdb.web.pasteur.fr/klebsiella/klebsiella.html), with a minimum of 94% query coverage and 94% identity. We used ABRicate (https://github.com/tseemann/abricate) to identify AMR genes based on the database from ResFinder (http://cge.cbs.dtu.dk/services/ResFinder).

### VAGE analyses

All the fully sequenced virulence plasmids containing *peg‐344* or *iucA* from GenBank (last accessed December 31, 2021) and newly sequenced virulence plasmids from our laboratory were used as references. The draft sequences of all virulence plasmids in our study were aligned using BLAST^61^ and custom *Perl* scripts. Their Inc groups were determined by performing BLAST against the plasmid replicon database, which included all plasmid replicon sequences downloaded from PlasmidFinder (https://cge.food.dtu.dk/services/PlasmidFinder/) as well as sequences collected by our laboratory. A threshold of ≥95% for both query coverage and identity was applied. Notably, in this study, we classified the IncFIB replicons into different types and subtypes based on the homology of their nucleotide sequences according to our previously published article^62^. Within each subtype, *repB*_IncFIB_ sequences showed ≥95% nucleotide identity, whereas <95% sequence identity was observed between different subtypes.

To ensure accuracy, we applied the following four criteria^41^: (1) the query coverage of the draft virulence plasmid sequence against the best‐match reference virulence plasmid was ≥70%; (2) the sequencing depth of contigs (>1000 bp) in the draft virulence plasmids was consistent; (3) the Inc group of the draft virulence plasmid matched that of the best‐match reference virulence plasmid; and (4) the draft virulence plasmids contained at least one of *peg‐344* and *iucA*.

The ICE*Kp* variants in 2097 hvKp strains were identified using Kleborate (https://github.com/klebgenomics/Kleborate). Additionally, the core genes *int* and *xis* were determined with thresholds of 100% query coverage and ≥99% identity. Given the high conservation of the sequences of Tn*7399* and *all*_island among hvKp isolates^21^, we extracted the reference sequences of Tn*7399* and *all*_island from the hvKp reference genome SGH10^21^. These sequences were then used to identify Tn*7399* and *all*_island in the 2097 isolates using BLAST.

The alignment rings of VAGEs from the complete genomes were visualized by BRIG^63^. The conjugation probabilities of complete virulence plasmids were identified using *oriTfinder* (https://tool-mml.sjtu.edu.cn/oriTfinder/oriTfinder.html).

### Construction of ML clustering trees and population structure analyses

Whole‐genome alignment of the 2097 hvKp isolates was performed to identify SNPs against the hvKp reference genome SGH10^21^ using Mummer v3.25^64^. Core genes were selected using Prokka^65^ and Roary^66^. SNPs located in the repetitive DNA regions (identified by RepeatMasker, http://www.repeatmasker.org/), and those in mobile genetic elements (including insertion sequences, transposons, integrons, and phage‐related genes) were excluded. Based on the remaining core SNPs, an ML clustering tree of the 2097 global hvKp isolates was constructed using IQ‐TREE V2.1.4^67^ with a bootstrap value of 1000. The phylogenetic tree was visualized using iTOL (https://itol.embl.de/).

To further verify the population structure of hvKp, we randomly selected one isolate from each ST and analyzed them using fineSTRUCTURE^68^. Clean reads from our sequenced 900 hvKp isolates were mapped to the SGH10 reference genome using Bowtie2^54^, with core SNPs identified using samtools^55^ and VarScan^69^. After filtering out all the SNPs in repetitive DNA regions and mobile genetic elements, an ML clustering tree of the 900 hvKp isolates was constructed using RAxML v.8.0.0^70^ based on the final core SNPs, with a bootstrap value of 100.

### Construction of recombination‐free phylogenetic trees

hvKp isolates of six CGs (CG23, CG258, CG65, CG86, CG412, and CG36) were subjected to sequence alignment and core SNPs detection, as described above for the 2097 hvKp isolates. We used ClonalFrameML^71^ to predict the recombination DNA regions and then remove all the SNP sites within these regions. We constructed the phylogenetic trees using the recombination‐free core SNPs of the hvKp isolates (non‐redundant) from the six CGs using RAxML v.8.0.0^70^ with a bootstrap value of 500, and visualized using iTOL.

### Bayesian phylogenetic inference and molecular dating analyses

Bayesian skyline analysis was conducted to estimate changes in the effective population size of 600 global nonredundant CG23 hvKp isolates and 319 global nonredundant CG258 hvKp isolates using BEAST v1.8.4^72^. Three standard substitution models—Hasegawa–Kishino–Yano (HKY), general time‐reversible (GTR), and Tamura‐Nei 93 (TN93)—were tested in combination with estimated/empirical base frequencies, gamma (G) site heterogeneity, and a relaxed molecular clock. After comparing all results, the “HKY + Estimated” model was selected as the best fit. The BEAST analysis was run with a Markov chain Monte Carlo length of 5 × 10^8^, sampling every 5 × 10^4^ steps. We visualized the resulting Bayesian skyline plot using Tracer v1.7^73^. Then, we constructed a time‐calibrated Bayesian MCC tree using *TreeAnnotator* (https://beast.community/treeannotator) and visualized using *FigTree* (http://tree.bio.ed.ac.uk/software/figtree/).

### Virulence phenotype assays

The hypermucoviscous phenotypes of our 900 sequenced isolates were determined using the string test. Isolates that formed strings longer than 5 mm when stretched with the tip of a sterile inoculation loop were defined as having a hypermucoviscous phenotype^42^. We further assessed the virulence levels of 329 representative isolates using the *Galleria mellonella* infection model^74^. *G. mellonella* larvae, weighing about 400–450 mg, were purchased from Tianjin Huiyude Biotech Company. Overnight cultures of the tested isolates were transferred to 15 ml of Brain–Heart Infusion (BHI) liquid medium at a ratio of 1:100 to achieve OD_600_ of 1.5. Then, the culture was further diluted and cultured to the exponential phase (OD_600_ = 1.0), and adjusted with sterile saline solution to a concentration of about 1 × 10^7^ CFU/ml. Each tested isolate corresponded to a group of 10 *G. mellonella* larvae. Each *G. mellonella* larva was infected with 10 μl of adjusted bacteria cultures, and incubated at 37°C for 72 h to observe the survival rate. All experiments were performed in triplicate. A known hvKp reference strain SGH10^21^, a classic *K. pneumoniae* strain, F726925, stored in our laboratory, and sterile saline solution were used as positive, negative, and blank controls, respectively.

## AUTHOR CONTRIBUTIONS

**Dongsheng Zhou:** Conceptualization, supervision, resources, writing—review and editing. **Fei Chen:** Conceptualization, supervision, writing—review and editing. **Xiaoyuan Jiang:** Writing—original draft, visualization, investigation, methodology, formal analysis, data curation. **Shuangshuang Li:** Writing—original draft, visualization, methodology, formal analysis. **Cuidan Li:** Writing—original draft, methodology. **Zhe Yin:** Writing—original draft, resources. **Fangzhou Chen:** Validation, resources. **Lingfei Hu:** Validation, resources. **Tianyu Lu:** Visualization, formal analysis. **Xiaoqiang Liu:** Formal analysis, data curation. **Yinyu Wang:** Validation, resources. **Guannan Ma:** Validation, resources. **Xiaoyu Wang:** Validation, resources.

## ETHICS STATEMENT

Not applicable.

## CONFLICT OF INTERESTS

The authors declare no conflict of interests.

## Supporting information

Table S1 and S8 are presented as separate Excel files containing additional data too large to fit in a PDF.

Supplemental materials. Figures S1 to S8 and Tables S2 to S7.

Table S1.

Table S8.

## Data Availability

The complete chromosome and plasmid sequences of the 177 hvKp isolates were submitted to GenBank with the accession numbers listed in Table S1. The 900 whole‐genome sequence data reported in this study have been uploaded to the Sequence Read Archive (SRA) database under BioProject PRJNA1147416. The code for phylogenetic analyses of hvKp is accessible at the github repository (https://github.com/ChenFLab/chenlab/blob/main/hvKp_code.txt).
