# Supplementary material for: The global genomic landscape of hypervirulent Klebsiella pneumoniae from 1932 to 2021
Source: mLife. 2025 Aug 24;4(4):378–96. doi: 10.1002/mlf2.70029 (PMC12395583; doi:10.1002/mlf2.70029)
Supplement: Supplementary file 1 — Supplemental materials. Figures S1 to S8 and Tables S2 to S7. [file MLF2-4-378-s001.pdf]

Supplemental information

The Global Genomic Landscape of Hypervirulent *Klebsiella pneumoniae* from 1932 to 2021

Xiaoyuan Jiang, Shuangshuang Li, Cuidan Li, Zhe Yin, Fangzhou Chen, Lingfei Hu, Tianyu Lu, Xiaoqiang Liu, Yinyu Wang, Guannan Ma, Xiaoyu Wang, Fei Chen, Dongsheng Zhou

This file includes: Figures S1 to S8 and Tables S2 to S7.

Table S1 and S8 are presented as separate Excel files containing additional data too large to fit in a PDF.

Supplemental information

Content

SUPPLEMENTARY FIGURES..3

Figure S1. Screening process of the 2,097 global hvKp isolates.. 3

Figure S2. Geographical and temporal distribution of different CGs in hvKp isolates......... 4

Figure S3. ML clustering tree of our 900 hvKp isolates sequenced in this study. 5

Figure S4. The hvKp population defined by *fineSTRUCTURE* analysis. 6

Figure S5. Major VGPs and VAGEs within the top six CGs.. 7

Figure S6. Alignment of VAGEs within each clade of the top six CGs. 8

Figure S6A. Alignments of the virulence plasmids and ICE*Kp* variants within CG23-Clade 1......8

Figure S6B. Alignments of the virulence plasmids and ICE*Kp* variants within CG23-Clade 2......8

Figure S6C. Alignments of the virulence plasmids and ICE*Kp* variants within CG258-Clade 1....9

Figure S6D. Alignments of the virulence plasmids and ICE*Kp* variants within CG258-Clade 2....9

Figure S6E. Alignments of the virulence plasmids and ICE*Kp* variants within CG258-Clade 3 ..10

Figure S6F. Alignment of the virulence plasmids within CG65-Clade 1 10

Figure S6G. Alignments of the virulence plasmids and ICE*Kp* variants within CG65-Clade 2....11

Figure S6H. Alignments of the virulence plasmids and ICE*Kp* variants within CG86-Clade 1....11

Figure S6I. Alignments of the virulence plasmids and ICE*Kp* variants within CG86-Clade 2 12

Figure S6J. Alignment of the virulence plasmids within CG412-Clade 1...................................12

Figure S6K. Alignment of the virulence plasmids within CG412-Clade 2..................................12

Figure S6L. Alignments of the virulence plasmids and ICE*Kp* variants within CG36-Clade 1 13

Figure S6M. Alignments of the virulence plasmids and ICE*Kp* variants within CG36-Clade 2 ...13

Figure S6N. Alignments of Tn*7399* and *all*_island 14

Figure S7. Bayesian skyline of the effective population size of CG23 hvKp isolates carrying the most prevalent VDC... 15

Figure S8. Virulence phenotypes of CG23 and CG258 hvKp isolates assessed using the *G. mellonella* infection model.. 16

SUPPLEMENTARY TABLES..17

Table S2. Inc groups of the virulence plasmids for the global 2,097 hvKp isolates. 17

Table S3. VAGEs on the chromosome for the 2,097 hvKp isolates. 19

Table S4A. Major VDCs within each clade of the top six CGs. 20

Table S4B. Major VDCs within the top six CGs. 21

Table S5. The proportions of MDR-hvKp and CR-hvKp/hv-CRKp within isolates of different CGs. 22

Table S6. Major virulent characteristics of carbapenem-resistance (CR) and carbapenem-sensitive (CS) hvKp isolates.. 23

Table S7. The positive rates in the string test for our 900 hvKp isolates............................ 24

Supplemental information

SUPPLEMENTARY FIGURES

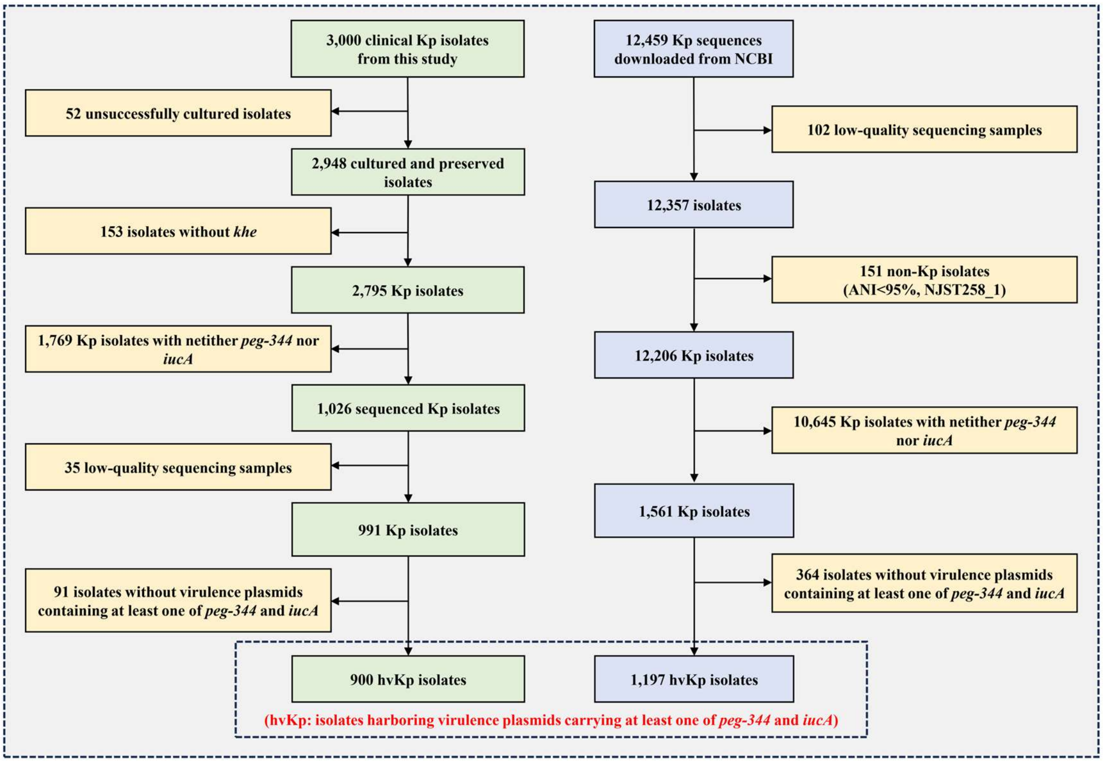

Figure S1. Screening process of the 2,097 global hvKp isolates. Kp: *Klebsiella pneumoniae*; hvKp: hypervirulent *K. pneumoniae*; NCBI: National Center for Biotechnology Information.

Supplemental information

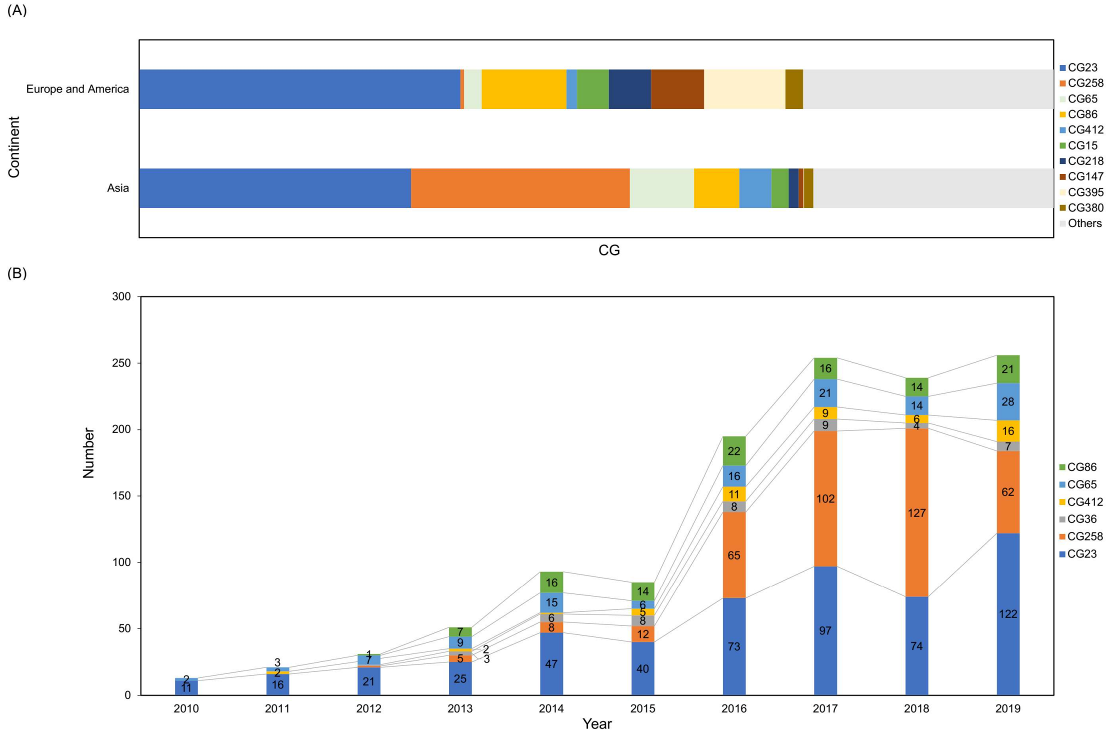

Figure S2. Geographical and temporal distribution of different CGs in hvKp isolates. (A) Bar plot representing the proportions of CGs in Europe and America, and Aisa. (B) Bar plot showing the number of hvKp isolates of the top six CGs (CG23, CG258, CG65, CG86, CG412, and CG36) from 2010 to 2019.

Supplemental information

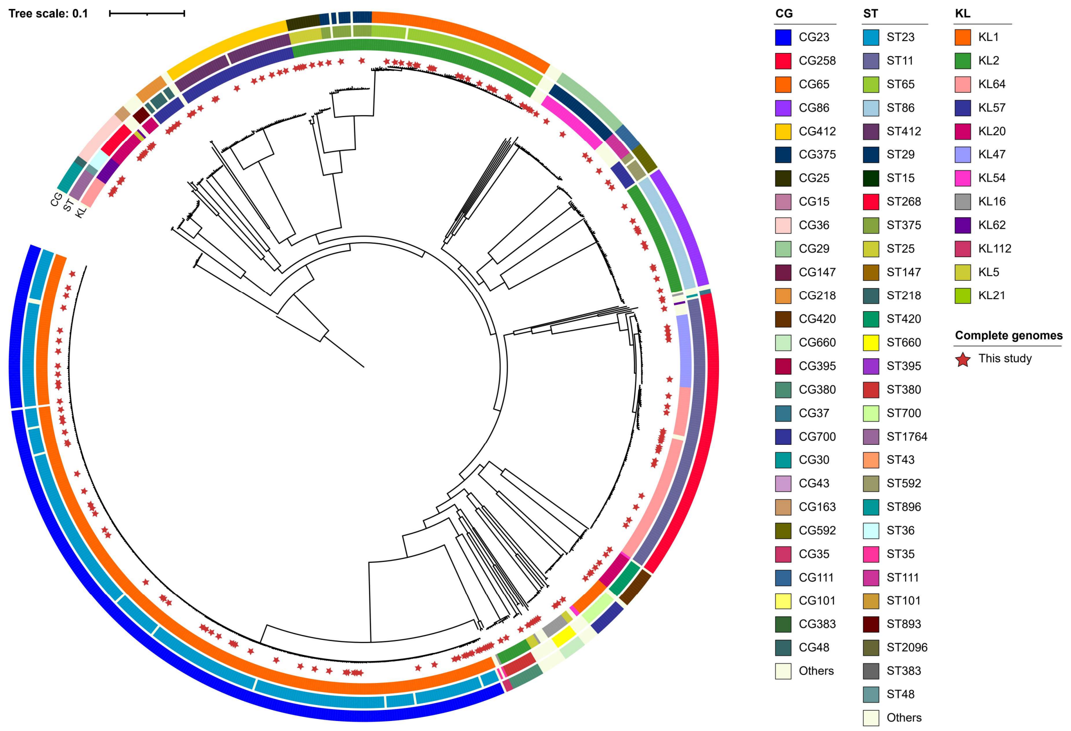

Figure S3. ML clustering tree of our 900 hvKp isolates sequenced in this study.
A ML clustering tree was constructed based on 503,605 core SNPs of our 900 hvKp isolates sequenced in this study. *K. variicola* isolate DSM 15968 was used as the outgroup but not shown in the tree. The circles from innermost to outermost in the tree indicate KLs, STs, and CGs, respectively. Isolates marked with red stars are the complete genomes sequenced in this study. CG, clonal group; ST, sequence type; KL, capsule locus; ML, maximum-likelihood; SNP, single nucleotide polymorphism.

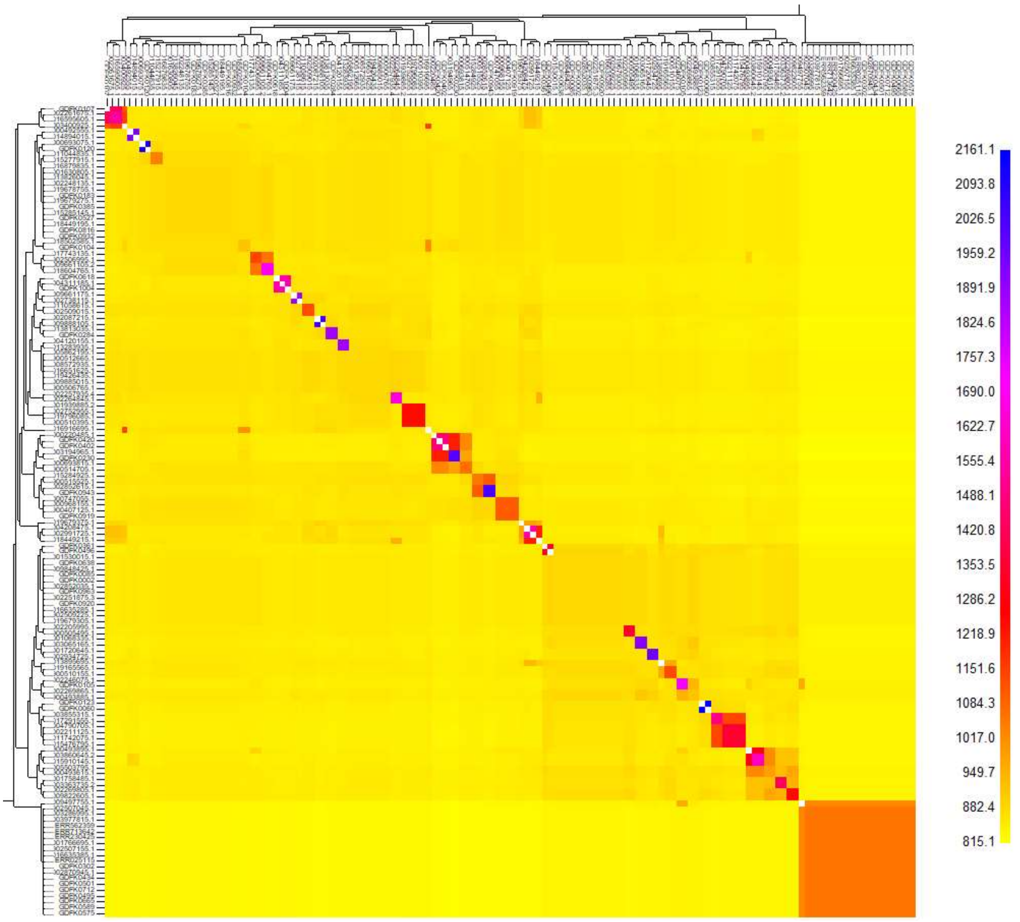

Figure S4. The hvKp population defined by *fineSTRUCTURE* analysis. The coancestry matrix was calculated based on 139 representative hvKp isolates from each ST. Columns represent donor isolates, while rows show recipient isolates. The grid colors indicate the number of sequence fragments that the donor has imported to the recipient. ST, sequence type.

Supplemental information

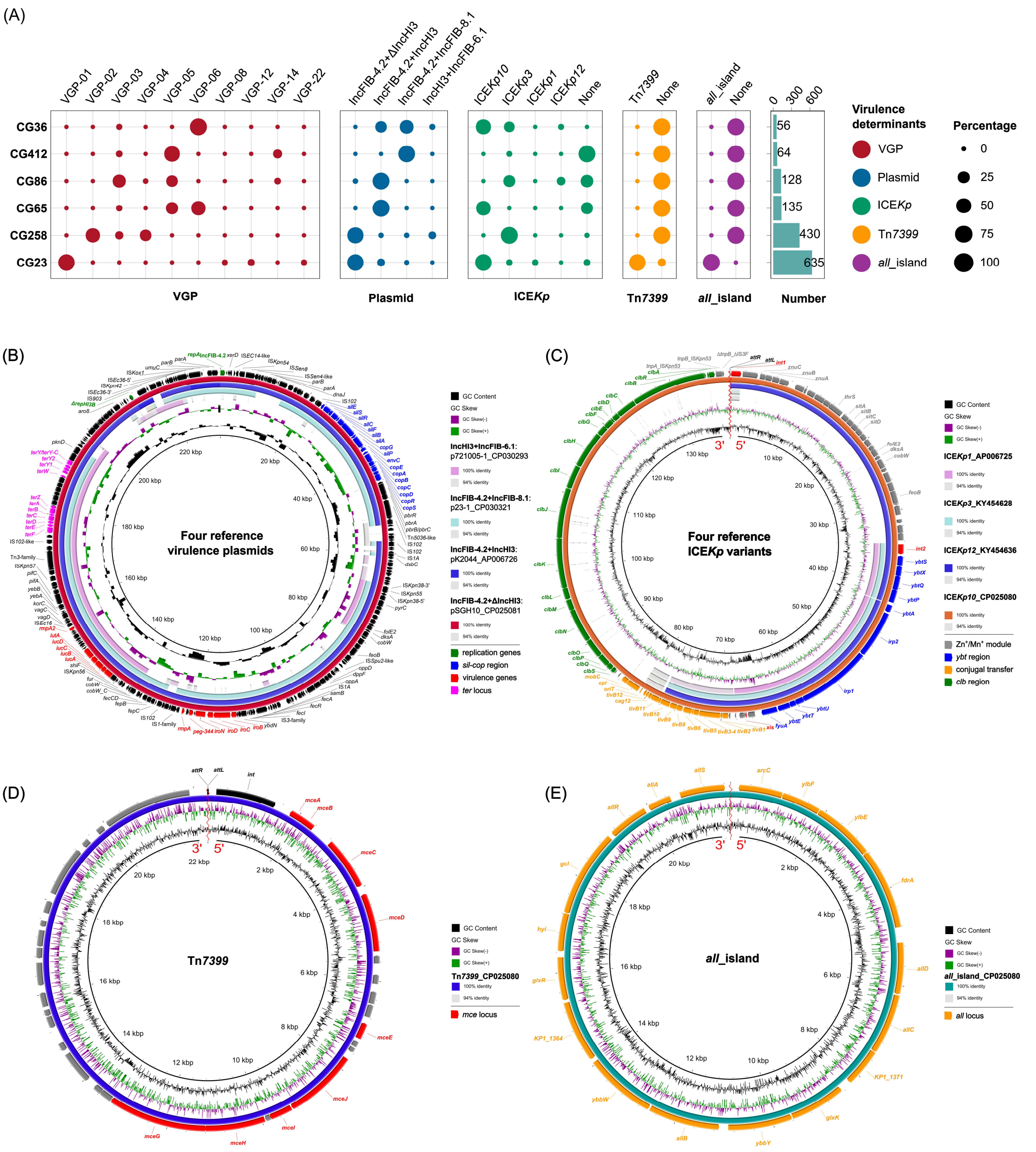

Figure S5. Major VGPs and VAGEs within the top six CGs. (A) Bubble charts showing the percentages of the major 10 VGPs, the top four Inc groups of virulence plasmids, the top four ICE*Kp* variants, Tn*7399*, and *all*_island within the top six CGs. The bar plot represented the number of isolates within the top six CGs. The size of the solid circles increases with the percentage. (B) Alignment of the four reference virulence plasmids (all aligned to pSGH10_CP025081). (C) Alignment of the four reference ICE*Kp* variants (all aligned to ICE*Kp10*_CP025080). (D) The molecular structure of Tn*7399*_CP025080. (E) The molecular structure of *all_island*_CP025080. Alignments were performed using BRIG with a sequence identity threshold of 94%. Genes are denoted by arrows and colored based on function classification. The three linear VAGEs (ICE*Kp*, Tn*7399*, and *all*_island) are marked with 5' and 3' in red. VGPs, virulence gene profiles; VAGEs, virulence-related accessory genetic elements; CGs, clonal groups.

Supplemental information

Figure S6. Alignment of VAGEs within each clade of the top six CGs. (A) to (M) represented the alignments of the virulence plasmids and ICE*Kp* variants within CG23-Clades 1/2, CG2258-Clades 1/2/3, CG65-Clades 1/2, CG86-Clades 1/2, CG412-Clades 1/2, and CG36-Clades 1/2, respectively. (N) represented the alignments of Tn*7399* and *all*_island. Alignments were performed using BRIG with a sequence identity threshold of 94%. Genes are denoted by arrows and are colored based on function classification. The three linear VAGEs (ICE*Kp*, Tn*7399*, and *all*_island) are marked with 5' and 3' in red. VAGEs, virulence-related accessory genetic elements; CGs, clonal groups.

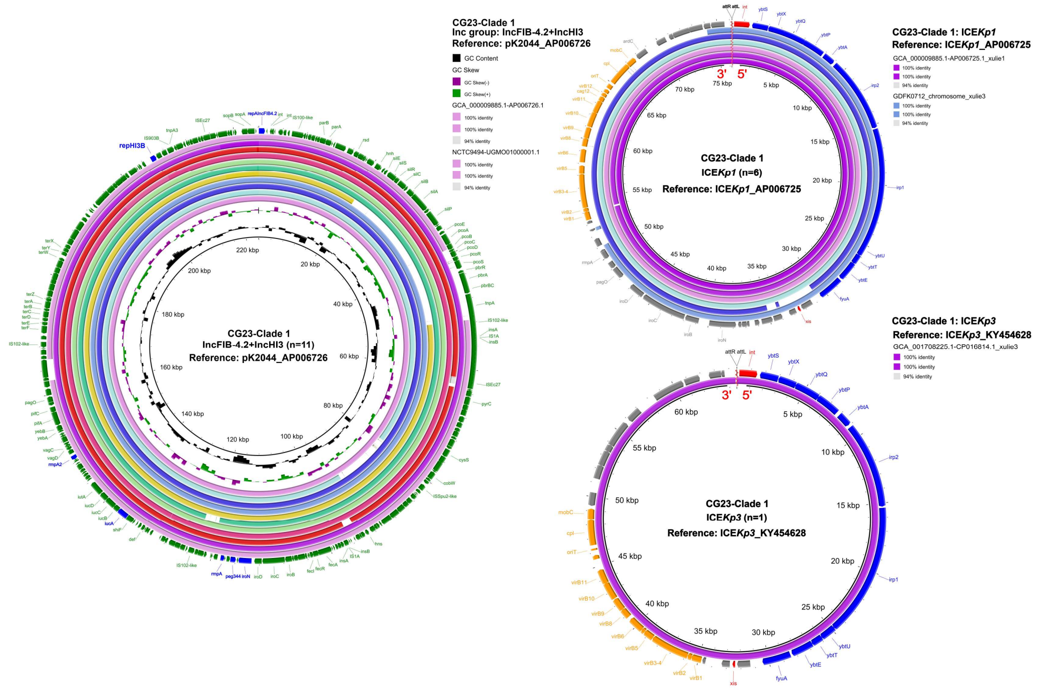

Figure S6A. Alignments of the virulence plasmids and ICE*Kp* variants within CG23-Clade 1

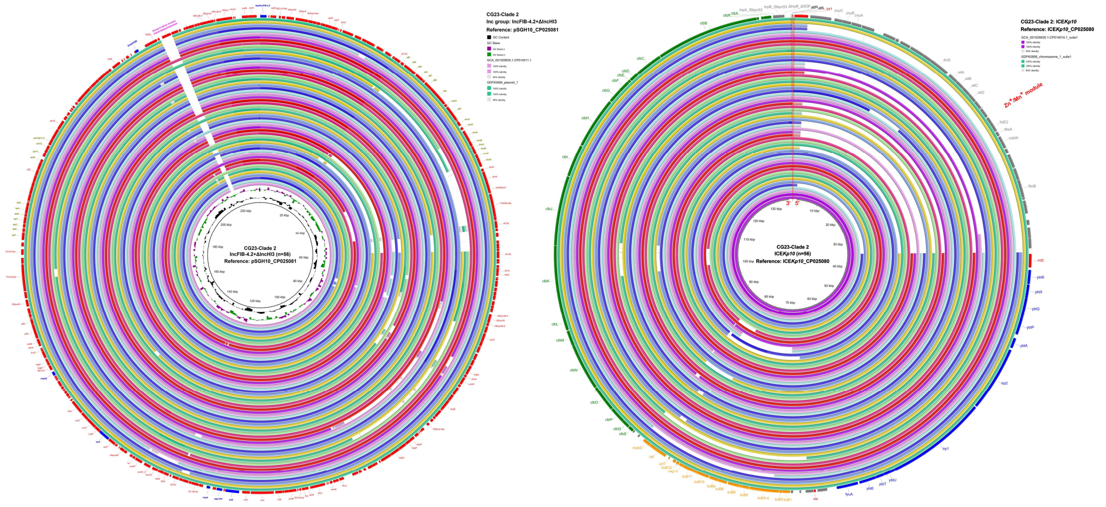

Figure S6B. Alignments of the virulence plasmids and ICE*Kp* variants within CG23-Clade 2

Supplemental information

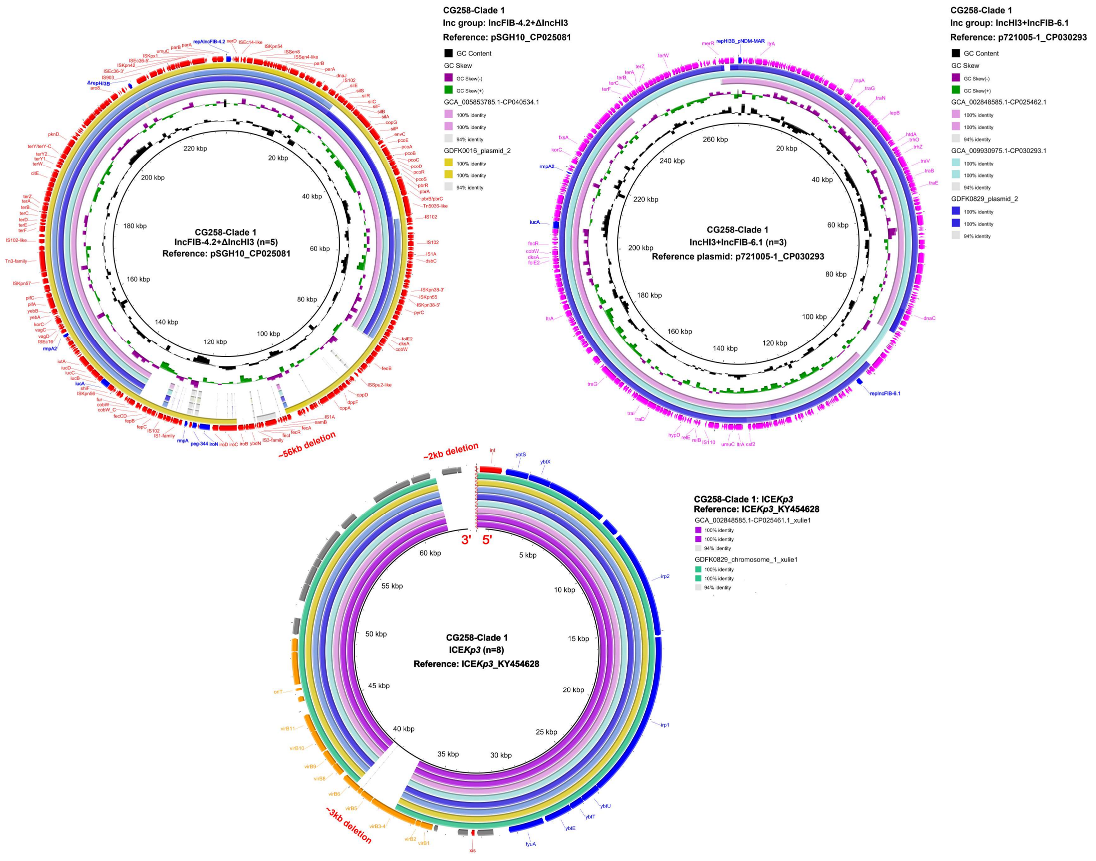

Figure S6C. Alignments of the virulence plasmids and ICE*Kp* variants within CG258-Clade 1

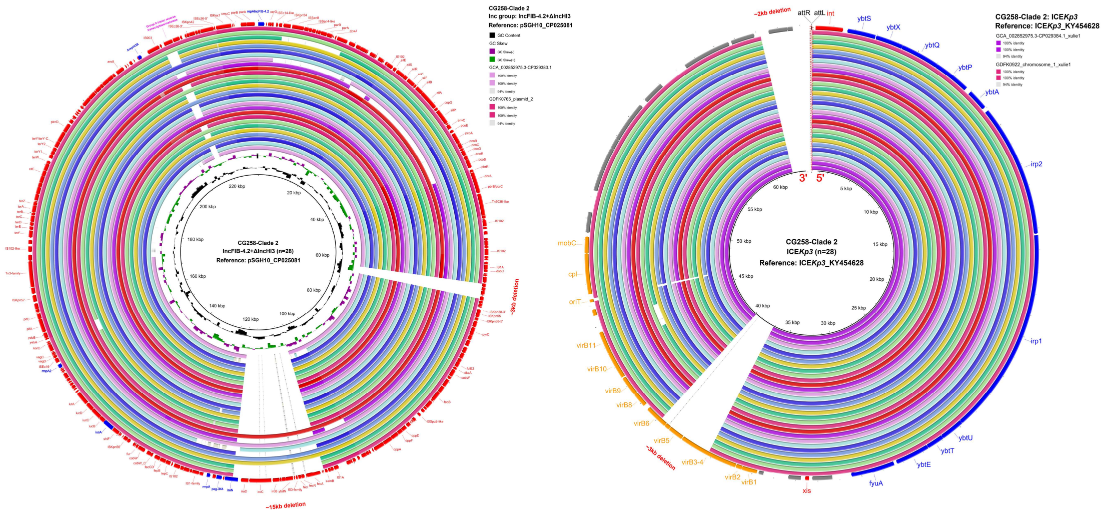

Figure S6D. Alignments of the virulence plasmids and ICE*Kp* variants within CG258-Clade 2

Supplemental information

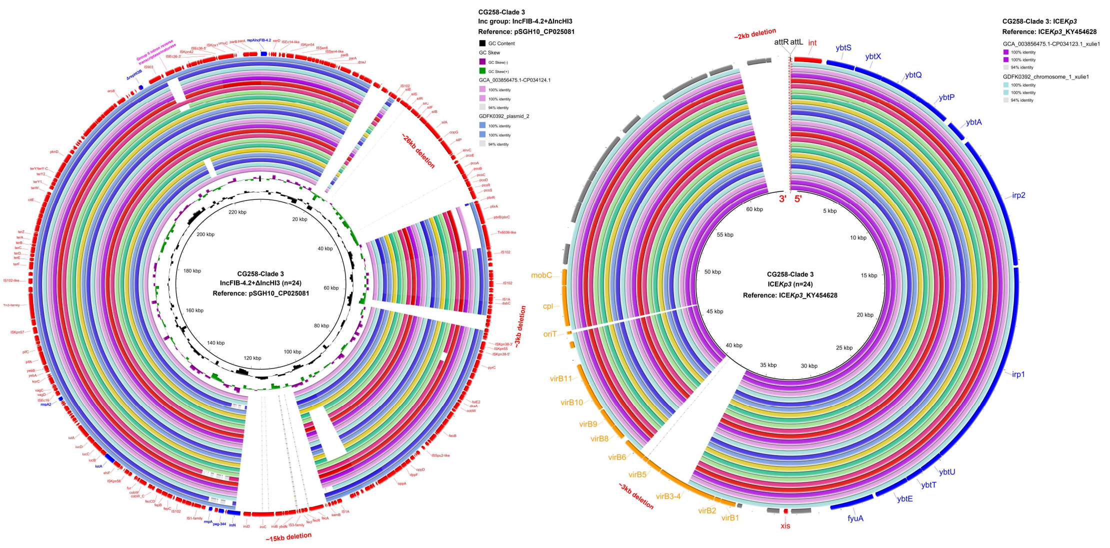

Figure S6E. Alignments of the virulence plasmids and ICE*Kp* variants within CG258-Clade 3

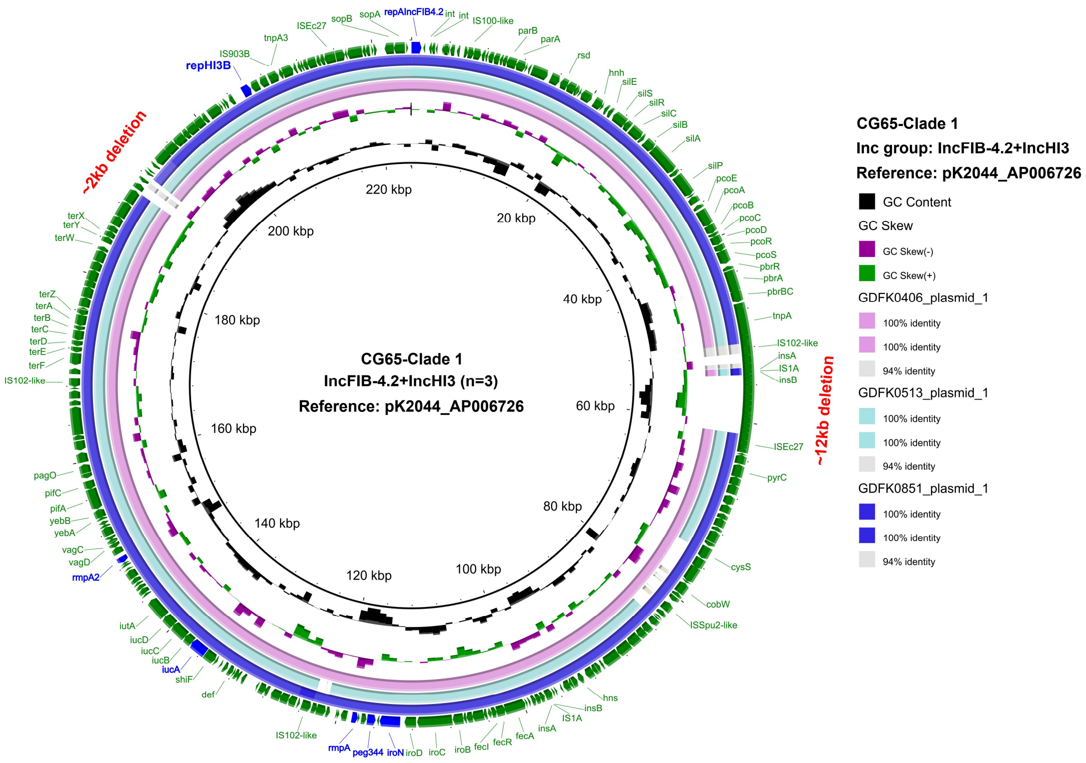

Figure S6F. Alignment of the virulence plasmids within CG65-Clade 1

Supplemental information

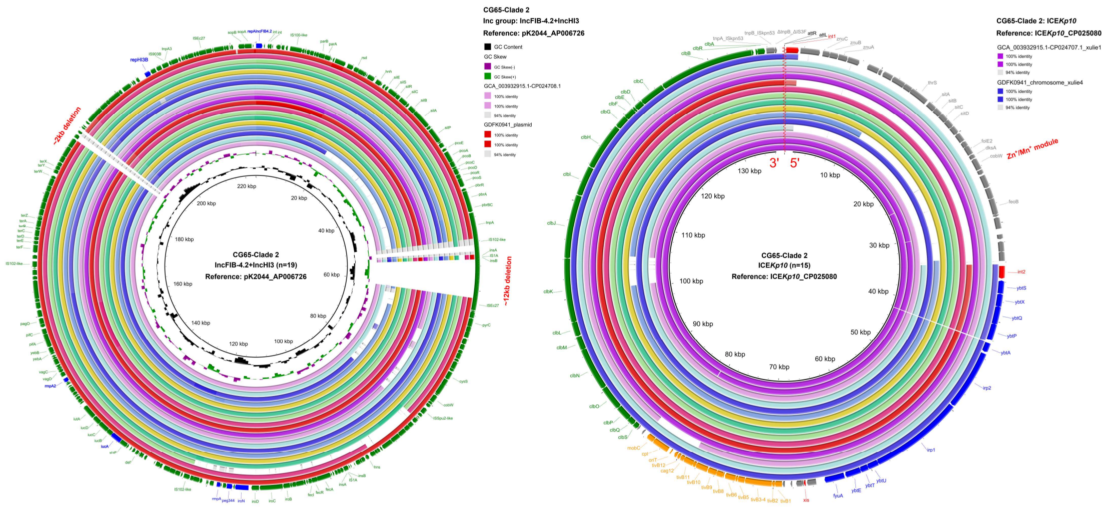

Figure S6G. Alignments of the virulence plasmids and ICE*Kp* variants within CG65-Clade 2

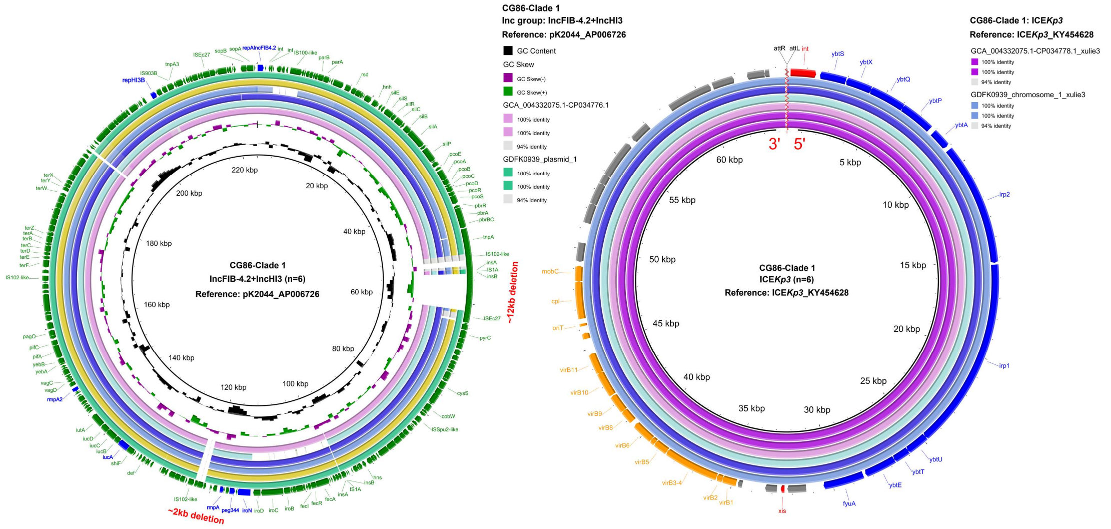

Figure S6H. Alignments of the virulence plasmids and ICE*Kp* variants within CG86-Clade 1

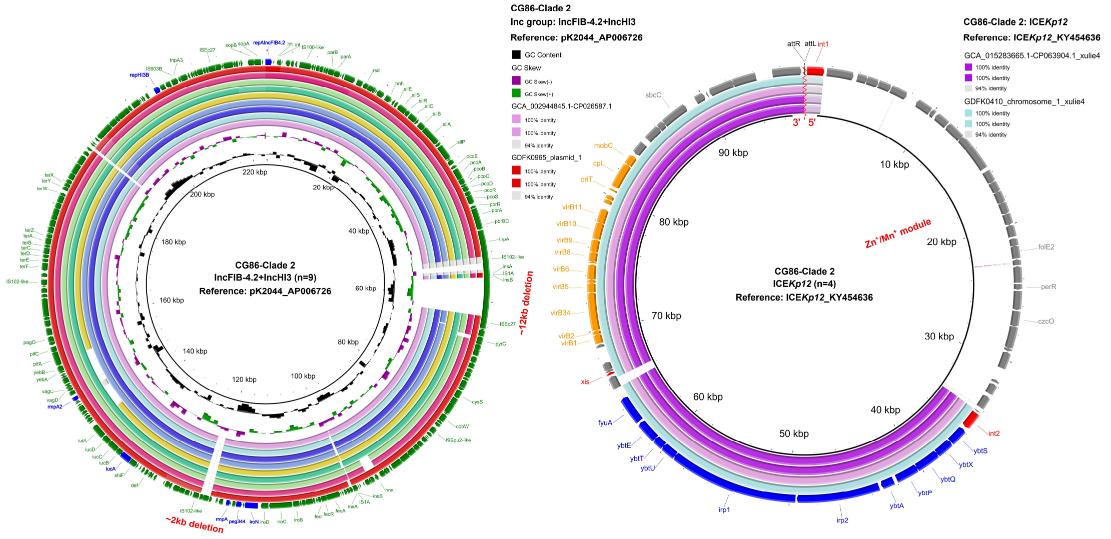

Supplemental information

Figure S6I. Alignments of the virulence plasmids and ICE*Kp* variants within CG86-Clade 2

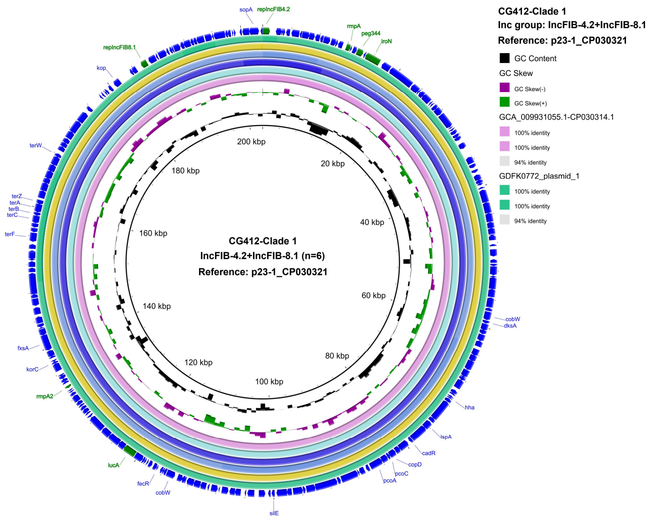

Figure S6J. Alignment of the virulence plasmids within CG412-Clade 1

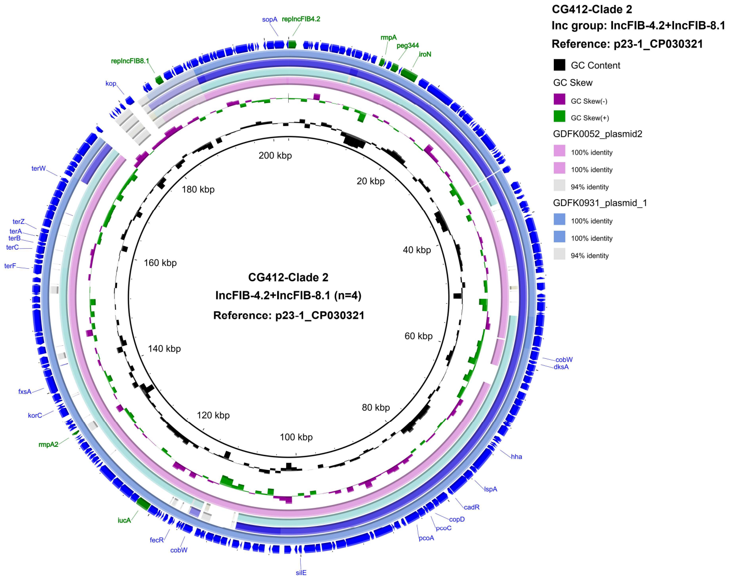

Figure S6K. Alignment of the virulence plasmids within CG412-Clade 2

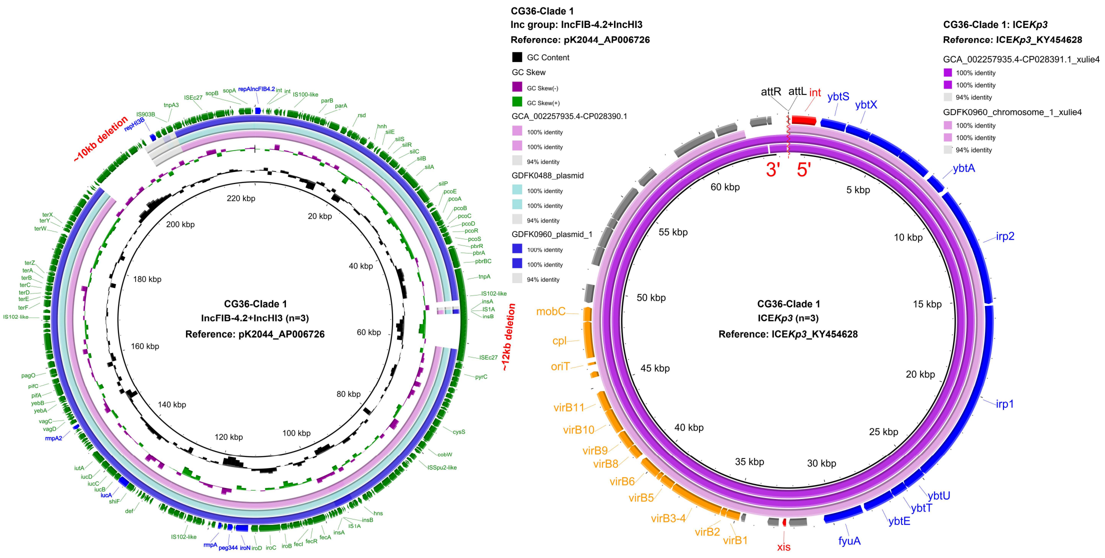

Figure S6L. Alignments of the virulence plasmids and ICE*Kp* variants within CG36-Clade 1

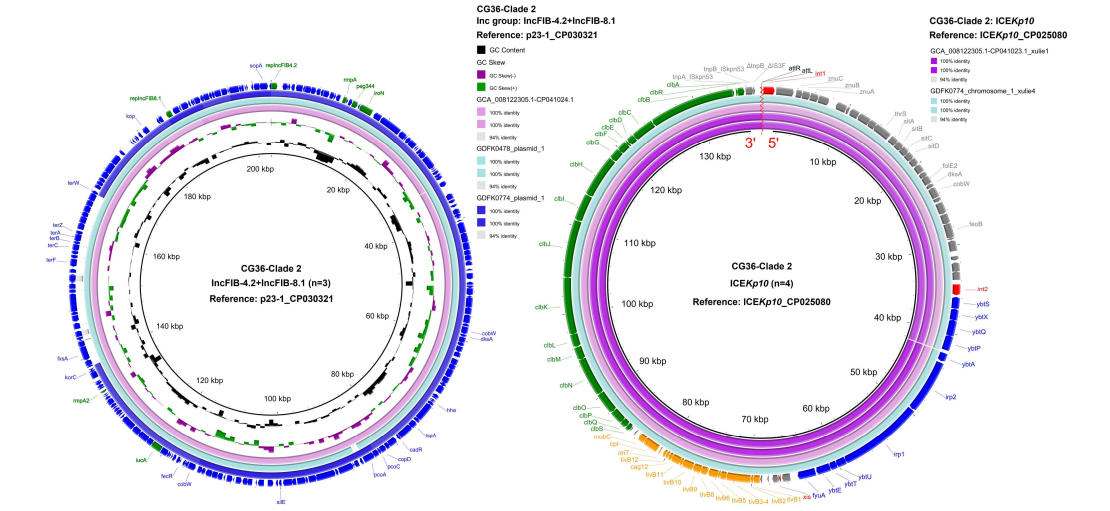

Figure S6M. Alignments of the virulence plasmids and ICE*Kp* variants within CG36-Clade 2

Supplemental information

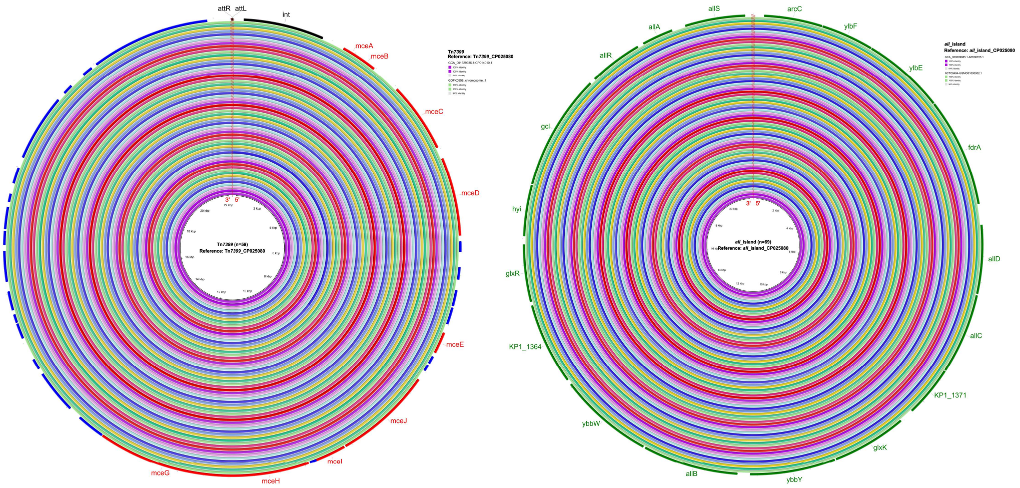

Figure S6N. Alignments of Tn*7399* and *all*_island

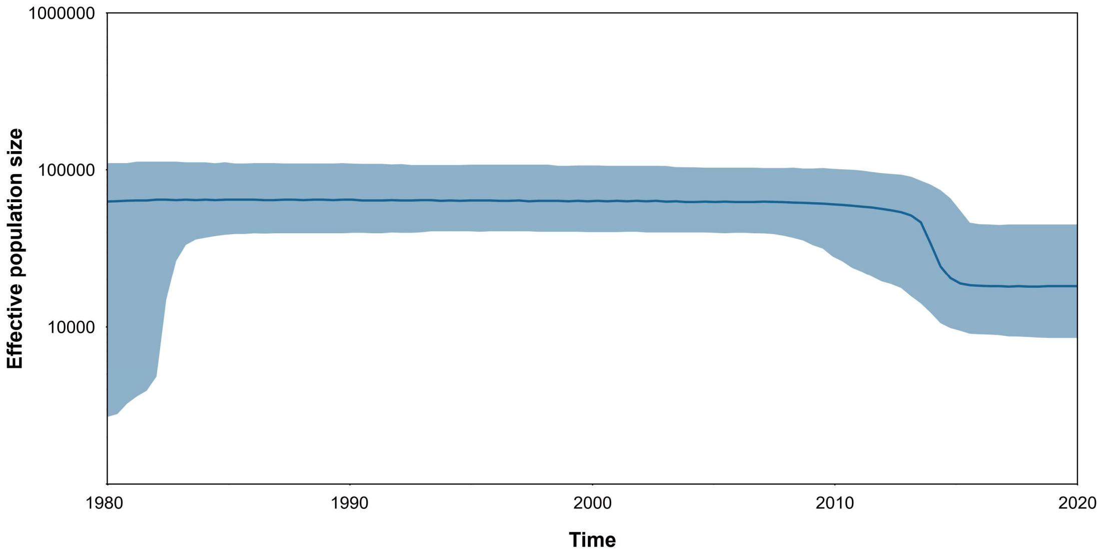

Figure S7. Bayesian skyline of the effective population size of CG23 hvKp isolates carrying the most prevalent VDC. A Bayesian skyline showed the effective population size of 472 non-redundant CG23 hvKp isolates with the most prevalent VDC of VGP-01–IncFIB-4.2+ΔIncHI3–ICE*Kp10*–Tn*7399*–*all*_island. Shadow region indicates the 95% probability density interval of the estimated population size. VDC, virulence determinant combination.

Supplemental information

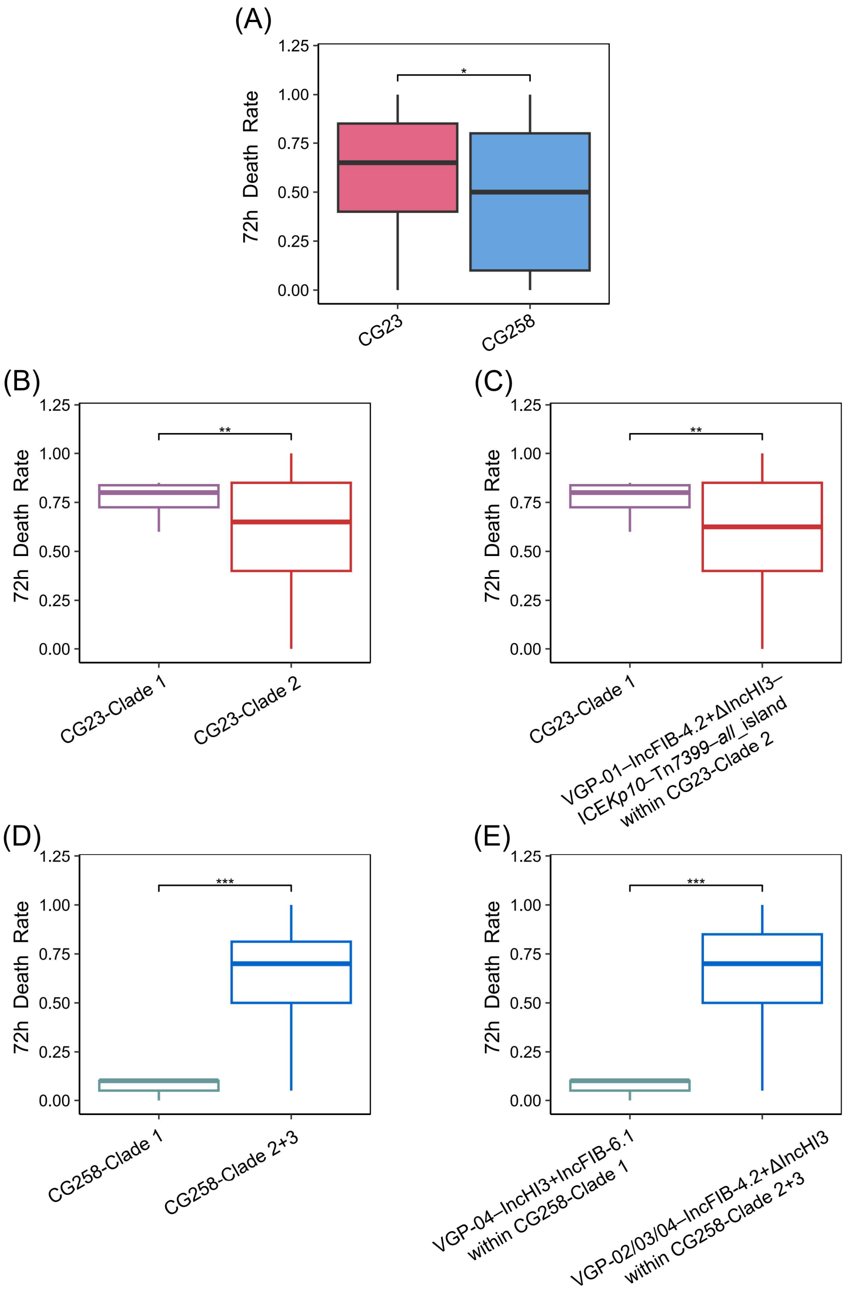

Figure S8. Virulence phenotypes of CG23 and CG258 hvKp isolates assessed using the *G. mellonella* infection model. Virulence was determined by the 72-hour mortality rates of *G. mellonella* larvae infected with hvKp isolates (1×10^7 CFU/mL). (A) Comparison of 72-hour mortality rates between CG23 and CG258 isolates. (B) Comparison of 72-hour mortality rates between CG23-Clade 1 and CG23-Clade 2 isolates. (C) Comparison of 72-hour mortality rates between CG23-Clade 1 isolates and CG23-Clade 2 isolates carrying the VDC of VGP-01–IncFIB-4.2+ΔIncHI3–ICE*Kp10*–Tn*7399*–*all*_island. (D) Comparison of 72-hour mortality rates between CG258-Clade 1 and CG258-Clades 2+3. (E) Comparison of 72-hour mortality rates between CG258-Clade 1 carrying the VDC of VGP-04–IncHI3+IncFIB-6.1 and CG258-Clades 2+3 carrying the VDC of VGP-02/03/04–IncFIB-4.2+ΔIncHI3. VDC, virulence determinant combination.

SUPPLEMENTARY TABLES

Table S2. Inc groups of the virulence plasmids for the global 2,097 hvKp isolates.

Inc group		Primary Inc replicon	Auxiliary Inc replicon	Truncated nonfunctional Inc replicon	Total (n=2,097) Number of plasmids/strains	%Percent	Rank
I	IncFIB-4.2 (n=1,717)	IncFIB-4.2	—	—	20	0.95	
		IncFIB-4.2	IncHI3	—	514	24.51	Top 2
		IncFIB-4.2	—	ΔIncHI3	952	45.40	Top 1
		IncFIB-4.2	IncFIB-8.1	—	218	10.40	Top 3
		IncFIB-4.2	—	ΔIncFIB-8.1	3	0.14	
		IncFIB-4.2	IncFIB-5.5	ΔIncHI3	1	0.05	
		IncFIB-4.2	IncR	ΔIncHI3	1	0.05	
		IncFIB-4.2	Inc_{pKPHS1}	ΔIncHI3	1	0.05	
		IncFIB-4.2	Inc_{pKPHS1}	ΔIncHI3+ΔInc_{pKPHS1}	1	0.05	
		IncFIB-4.2	$IncFII_K$+$Inc_{pA1763\text{-}KPC}$	—	1	0.05	
		IncFIB-4.2	$IncFII_K$+$Inc_{pA1763\text{-}KPC}$	ΔIncHI3	1	0.05	
		IncFIB-4.2	$IncFII_{pKp\_Goe\_414\text{-}4}$	ΔIncHI3	4	0.19	
II	IncFIB-4.1 (n=200)	IncFIB-4.1	—	—	9	0.43	
		IncFIB-4.1	—	ΔIncHI3	1	0.05	
		IncFIB-4.1	$IncFII_K$	—	97	4.63	Top 5
		IncFIB-4.1	—	$\Delta IncFII_K$	2	0.10	
		IncFIB-4.1	$IncFII_{pKDO1}$	—	59	2.81	

		IncFIB-4.1	—	ΔIncFII$_{pKDO1}$	31	1.48	
		IncFIB-4.1	IncFII$_{K}$+IncFII$_{pKDO1}$	—	1	0.05	
**III**	**IncFIB-6.1 (n=12)**	IncFIB-6.1	—	—	1	0.05	
		IncFIB-6.1	—	ΔIncHI3	2	0.10	
		IncFIB-6.1	IncFIB-8.1	—	7	0.33	
		IncFIB-6.1	—	ΔIncFIB-8.1	1	0.05	
		IncFIB-6.1	IncFIB-8.1	ΔIncFIB-6.1	1	0.05	
**IV**	**IncFIB-8.1 (n=7)**	IncFIB-8.1	—	—	3	0.14	
		IncFIB-8.1	—	ΔIncFIB-4.2	1	0.05	
		IncFIB-8.1	—	ΔIncFIB-6.1	3	0.14	
**V**	**IncFIB-7.1 (n=2)**	IncFIB-7.1	IncFII$_{K}$	—	1	0.05	
		IncFIB-7.1	IncFII$_{pKDO1}$	—	1	0.05	
**VI**	**IncHI3 (n=159)**	IncHI3	—	—	26	1.24	
		IncHI3	IncFIB-6.1	—	98	4.67	Top 4
		IncHI3	—	ΔIncFIB-6.1	35	1.67	
**Total**					2,097	100.00	1,814 (86.50%)

Note: Inc group, incompatibility group.

Supplemental information

Table S3. VAGEs on the chromosome for the 2,097 hvKp isolates.

**VAGEs**		**Total (n=2,097)**	
		**Number of strains**	**%Percent**
**ICE*Kp***	ICE*Kp10*	734	35.00
	ICE*Kp3*	629	30.00
	ICE*Kp12*	82	3.91
	ICE*Kp1*	57	2.72
	ICE*Kp4*	47	2.24
	ICE*Kp6*	27	1.29
	ICE*Kp5*	24	1.14
	ICE*Kp16*	12	0.57
	ICE*Kp11*	9	0.43
	ICE*Kp2*	2	0.10
	None	474	22.60
**Tn*7399***	Tn*7399*	616	29.38
	None	1,481	70.62
***all*_island**	*all*_island	676	32.24
	None	1,421	67.76

Note: VAGEs, virulence-related accessory genetic elements.

Supplemental information

Table S4A. Major VDCs within each clade of the top six CGs.

CG	Clade	Major VDCs	Number	Percent% (per Clade)
**CG23 (n=635)**	**Clade-1 (n=47)**	VGP-12–IncFIB-4.2+IncHI3–ICE*Kp1*–None–*all*_island	14	29.79
		VGP-12–IncFIB-4.2+IncHI3–ICE*Kp3*–None–*all*_island	10	21.28
		VGP-22–IncFIB-4.2+IncHI3–None–Tn*7399*–*all*_island	9	19.15
		VGP-08–IncFIB-4.2+IncHI3–None–None–*all*_island	8	17.02
	**Clade-2 (n=588)**	VGP-01–IncFIB-4.2+ΔIncHI3–ICE*Kp10*–Tn*7399*–*all*_island	498	84.69
**CG258 (n=430)**	**Clade-1 (n=77)**	VGP-04–IncHI3+IncFIB-6.1–ICE*Kp3*–None–None	24	31.17
		VGP-04–IncFIB-4.2+ΔIncHI3–ICE*Kp3*–None–None	22	28.57
		VGP-03–IncFIB-4.2+ΔIncHI3–ICE*Kp3*–None–None	11	14.29
	**Clade-2 (n=237)**	VGP-02–IncFIB-4.2+ΔIncHI3–ICE*Kp3*–None–None	164	69.20
		VGP-04–IncFIB-4.2+ΔIncHI3–ICE*Kp3*–None–None	29	12.24
		VGP-03–IncFIB-4.2+ΔIncHI3–ICE*Kp3*–None–None	24	10.13
	**Clade-3 (n=114)**	VGP-02–IncFIB-4.2+ΔIncHI3–ICE*Kp3*–None–None	77	67.54
		VGP-04–IncFIB-4.2+ΔIncHI3–ICE*Kp3*–None–None	27	23.68
**CG65 (n=135)**	**Clade-1 (n=36)**	VGP-05–IncFIB-4.2+IncHI3–None–None–None	33	91.67
	**Clade-2 (n=99)**	VGP-06–IncFIB-4.2+IncHI3–ICE*Kp10*–None–None	75	75.76
		VGP-05–IncFIB-4.2+IncHI3–None–None–None	13	13.13
**CG86 (n=128)**	**Clade-1 (n=49)**	VGP-03–IncFIB-4.2+IncHI3–ICE*Kp3*–None–None	38	77.55
	**Clade-2 (n=78)**	VGP-05–IncFIB-4.2+IncHI3–None–None–None	34	43.59
		VGP-03–IncFIB-4.2+IncHI3–ICE*Kp12*–None–None	14	17.95
**CG412 (n=64)**	**Clade-1 (n=29)**	VGP-05–IncFIB-4.2+IncFIB-8.1–None–None–None	23	79.31
	**Clade-2 (n=35)**	VGP-05–IncFIB-4.2+IncFIB-8.1–None–None–None	21	60.00
		VGP-14–IncFIB-4.2+IncFIB-8.1–None–None–None	7	20.00
**CG36 (n=56)**	**Clade-1 (n=14)**	VGP-06–IncFIB-4.2+IncHI3–ICE*Kp3*–None–None	14	100.00
	**Clade-2 (n=41)**	VGP-06–IncFIB-4.2+IncFIB-8.1–ICE*Kp10*–None–None	30	73.17

Note: VDCs with a proportion of ≥10% and a minimum of 5 strains within each clade of the top six CGs were retained. A total of 17 non-redundant VDCs were preserved across the top six CGs and their 13 clades. Each VDC consists of sequentially arranged virulence determinants: VGP, virulence plasmid, Tn*7399*, and *all*_island. "None" indicates the absence of the corresponding virulence determinant. VDC, virulence determinant combination; VGP, virulence gene profile; CG, clonal group.

Supplemental information

Table S4B. Major VDCs within the top six CGs.

CG	Major VDCs	Number	Percent% (per CG)
**CG23 (n=635)**	VGP-01–IncFIB-4.2+ΔIncHI3–ICE*Kp10*–Tn*7399*–*all*_island	498	78.43
	VGP-12–IncFIB-4.2+IncHI3–ICE*Kp1*–None–*all*_island	14	2.20
	VGP-12–IncFIB-4.2+IncHI3–ICE*Kp3*–None–*all*_island	10	1.57
	VGP-22–IncFIB-4.2+IncHI3–None–Tn*7399*–*all*_island	9	1.42
	VGP-08–IncFIB-4.2+IncHI3–None–None–*all*_island	8	1.26
**CG258 (n=430)**	VGP-02–IncFIB-4.2+ΔIncHI3–ICE*Kp3*–None–None	241	56.05
	VGP-04–IncFIB-4.2+ΔIncHI3–ICE*Kp3*–None–None	78	18.14
	VGP-03–IncFIB-4.2+ΔIncHI3–ICE*Kp3*–None–None	35	8.14
	VGP-04–IncHI3+IncFIB-6.1–ICE*Kp3*–None–None	24	5.58
**CG65 (n=135)**	VGP-06–IncFIB-4.2+IncHI3–ICE*Kp10*–None–None	75	55.56
	VGP-05–IncFIB-4.2+IncHI3–None–None–None	46	34.07
**CG86 (n=128)**	VGP-03–IncFIB-4.2+IncHI3–ICE*Kp3*–None–None	38	29.69
	VGP-05–IncFIB-4.2+IncHI3–None–None–None	34	26.56
	VGP-03–IncFIB-4.2+IncHI3–ICE*Kp12*–None–None	14	10.94
**CG412 (n=64)**	VGP-05–IncFIB-4.2+IncFIB-8.1–None–None–None	44	68.75
	VGP-14–IncFIB-4.2+IncFIB-8.1–None–None–None	7	10.94
**CG36 (n=56)**	VGP-06–IncFIB-4.2+IncHI3–ICE*Kp3*–None–None	14	25.00
	VGP-06–IncFIB-4.2+IncFIB-8.1–ICE*Kp10*–None–None	30	53.57

Note: A total of 17 non-redundant VDCs were preserved across the top six CGs. Each VDC consists of sequentially arranged virulence determinants: VGP, virulence plasmid, Tn*7399*, and *all*_island. "None" indicates the absence of the corresponding virulence determinant. VDC, virulence determinant combination; VGP, virulence gene profile; CG, clonal group.

Supplemental information

Table S5. The proportions of MDR-hvKp and CR-hvKp/hv-CRKp within isolates of different CGs.

	Number	MDR-hvKp Number (%Percent)	CR-hvKp/hv-CRKp Number (%Percent)
**Overall**	2,097	872 (41.6)	698 (33.3)
**CG23**	635	60 (9.4)	40 (6.3)
**CG258**	430	416 (96.7)	417 (97.0)
**CG65**	135	18 (13.3)	18 (13.3)
**CG86**	128	13 (10.2)	13 (10.2)
**CG412**	64	4 (6.2)	2 (3.1)
**CG36**	56	19 (33.9)	19 (33.9)
**CG29**	47	2 (4.3)	2 (4.3)
**CG15**	42	41 (97.6)	36 (85.7)
**CG375**	36	1 (2.8)	1 (2.8)
**CG25**	34	18 (52.9)	9 (26.5)
**CG147**	31	30 (96.8)	27 (87.1)
**CG218**	31	9 (29.0)	9 (29.0)
**CG420**	31	10 (32.3)	5 (16.1)
**CG660**	28	6 (21.4)	3 (10.7)
**CG395**	24	24 (100.0)	23 (95.8)
**CG380**	23	0 (0.0)	0 (0.0)
**CG37**	22	22 (100.0)	1 (4.5)
**CG700**	21	21 (100.0)	0 (0.0)
**CG30**	19	0 (0.0)	0 (0.0)
**CG43**	18	18 (100.0)	3 (16.7)
**CG592**	17	1 (5.9)	1 (5.9)
**CG163**	15	7 (46.7)	0 (0.0)
**CG35**	15	13 (86.7)	0 (0.0)
**CG2096**	14	14 (100.0)	8 (57.1)
**CG111**	13	0 (0.0)	0 (0.0)
**CG101**	12	10 (83.3)	10 (83.3)
**CG383**	10	10 (100.0)	10 (100.0)
**CG48**	10	10 (100.0)	6 (60.0)
**others**	136	75 (55.1)	35 (25.7)
p		**< 0.001**	**< 0.001**

Note: MDR, multidrug resistance; CR, carbapenem resistance.

Supplemental information

Table S6. Major virulent characteristics of carbapenem-resistance (CR) and carbapenem-sensitive (CS) hvKp isolates.

**hvKp**	**CG**	**VGP**	**Plasmid**	**ICE*Kp***	**Tn*7399***	***all*_island**	**Number**	**%Percent**	**Rank**
**CR (n=698)**	CG258	VGP-02	IncFIB-4.2+ΔIncHI3	ICE*Kp3*	–	–	232	33.24	Top01
	CG258	VGP-04	IncFIB-4.2+ΔIncHI3	ICE*Kp3*	–	–	78	11.17	Top02
	CG258	VGP-03	IncFIB-4.2+ΔIncHI3	ICE*Kp3*	–	–	35	5.01	Top03
	CG258	VGP-04	IncHI3+IncFIB-6.1	ICE*Kp3*	–	–	28	4.01	Top04
	CG15	VGP-04	IncFIB-4.2+IncHI3	ICE*Kp12*	–	–	26	3.72	Top05
**CS (n=1399)**	CG23	VGP-01	IncFIB-4.2+ΔIncHI3	ICE*Kp10*	Tn*7399*	*all*_island	473	33.81	Top01
	CG65	VGP-06	IncFIB-4.2+IncHI3	ICE*Kp10*	–	–	63	4.50	Top02
	CG65	VGP-05	IncFIB-4.2+IncHI3	–	–	–	44	3.15	Top03
	CG412	VGP-05	IncFIB-4.2+IncFIB-8.1	–	–	–	43	3.07	Top04
	CG86	VGP-05	IncFIB-4.2+IncHI3	–	–	–	35	2.50	Top05

Supplemental information

Table S7. The positive rates in the string test for our 900 hvKp isolates.

CG	Positive		Total
	Number	%Percent	
**CG23**	277	80.99	342
**CG258**	10	8.20	122
**CG65**	70	88.61	79
**CG412**	45	83.33	54
**CG86**	48	92.31	52
**CG29**	24	72.73	33
**CG36**	11	47.83	23
**CG375**	22	100.00	22
**CG420**	11	68.75	16
**CG700**	15	100.00	15
**CG380**	14	100.00	14
**CG25**	12	85.71	14
**CG218**	12	92.31	13
**CG30**	10	76.92	13
**CG592**	12	100.00	12
**CG111**	9	81.82	11
**CG660**	2	20.00	10
**CG163**	1	20.00	5
**CG1049**	3	75.00	4
**CG35**	0	0.00	3
**CG828**	3	100.00	3
**CG48**	0	0.00	3
**CG5**	1	50.00	2
**CG889**	0	0.00	2
**CG37**	0	0.00	2
**CG24**	0	0.00	2
**CG2058**	2	100.00	2
**CG685**	0	0.00	1
**CG5020**	0	0.00	1
**CG55**	1	100.00	1
**CG76**	0	0.00	1
**CG661**	0	0.00	1
**CG39**	1	100.00	1
**CG806**	0	0.00	1
**CG5405**	1	100.00	1
**CG314**	1	100.00	1
**CG1265**	1	100.00	1
**CG202**	1	100.00	1
**CG882**	1	100.00	1
**CG2846**	1	100.00	1
**CG1117**	0	0.00	1
**Singleton**	7	53.85	13
**Total**	629	69.89	900
